# Discovery of the Pseudomonas Polyyne Protegencin by a Phylogeny-Guided Study of Polyyne Biosynthetic Gene Cluster Diversity

**DOI:** 10.1128/mBio.00715-21

**Published:** 2021-08-03

**Authors:** Alex J. Mullins, Gordon Webster, Hak Joong Kim, Jinlian Zhao, Yoana D. Petrova, Christina E. Ramming, Matthew Jenner, James A. H. Murray, Thomas R. Connor, Christian Hertweck, Gregory L. Challis, Eshwar Mahenthiralingam

**Affiliations:** a Microbiomes, Microbes and Informatics Group, Organisms and Environment Division, School of Biosciences, Cardiff University, Cardiff, United Kingdom; b Department of Biomolecular Chemistry, Leibniz Institute for Natural Product Research and Infection Biology, Hans Knöll Institute, Jena, Germany; c Department of Chemistry, University of Warwick, Coventry, United Kingdom; d Warwick Integrative Synthetic Biology Centre, University of Warwick, Coventry, United Kingdom; e Molecular Biosciences Division, School of Biosciences, Cardiff University, Cardiff, United Kingdom; f Faculty of Biological Sciences, Friedrich Schiller University Jena, Jena, Germany; g Department of Biochemistry and Molecular Biology, Biomedicine Discovery Institute, Monash University, Clayton, Victoria, Australia; h ARC Centre of Excellence for Innovations in Peptide and Protein Science, Monash University, Clayton, Victoria, Australia; University of Pittsburgh

**Keywords:** *Pseudomonas*, biosynthetic gene clusters, natural products, phylogenetics, polyynes

## Abstract

Natural products that possess alkyne or polyyne moieties have been isolated from a variety of biological sources and possess a broad a range of bioactivities. In bacteria, the basic biosynthesis of polyynes is known, but their biosynthetic gene cluster (BGC) distribution and evolutionary relationship to alkyne biosynthesis have not been addressed. Through comprehensive genomic and phylogenetic analyses, the distribution of alkyne biosynthesis gene cassettes throughout bacteria was explored, revealing evidence of multiple horizontal gene transfer events. After investigation of the evolutionary connection between alkyne and polyyne biosynthesis, a monophyletic clade was identified that possessed a conserved seven-gene cassette for polyyne biosynthesis that built upon the conserved three-gene cassette for alkyne biosynthesis. Further diversity mapping of the conserved polyyne gene cassette revealed a phylogenetic subclade for an uncharacterized polyyne BGC present in several Pseudomonas species, designated *pgn*. Pathway mutagenesis and high-resolution analytical chemistry showed the Pseudomonas protegens *pgn* BGC directed the biosynthesis of a novel polyyne, protegencin. Exploration of the biosynthetic logic behind polyyne production, through BGC mutagenesis and analytical chemistry, highlighted the essentiality of a triad of desaturase proteins and a thioesterase in both the P. protegens *pgn* and Trinickia caryophylli (formerly Burkholderia caryophylli) caryoynencin pathways. We have unified and expanded knowledge of polyyne diversity and uniquely demonstrated that alkyne and polyyne biosynthetic gene clusters are evolutionarily related and widely distributed within bacteria. The systematic mapping of conserved biosynthetic genes across the available bacterial genomic diversity proved to be a fruitful method for discovering new natural products and better understanding polyyne biosynthesis.

## INTRODUCTION

**FIG 1:**
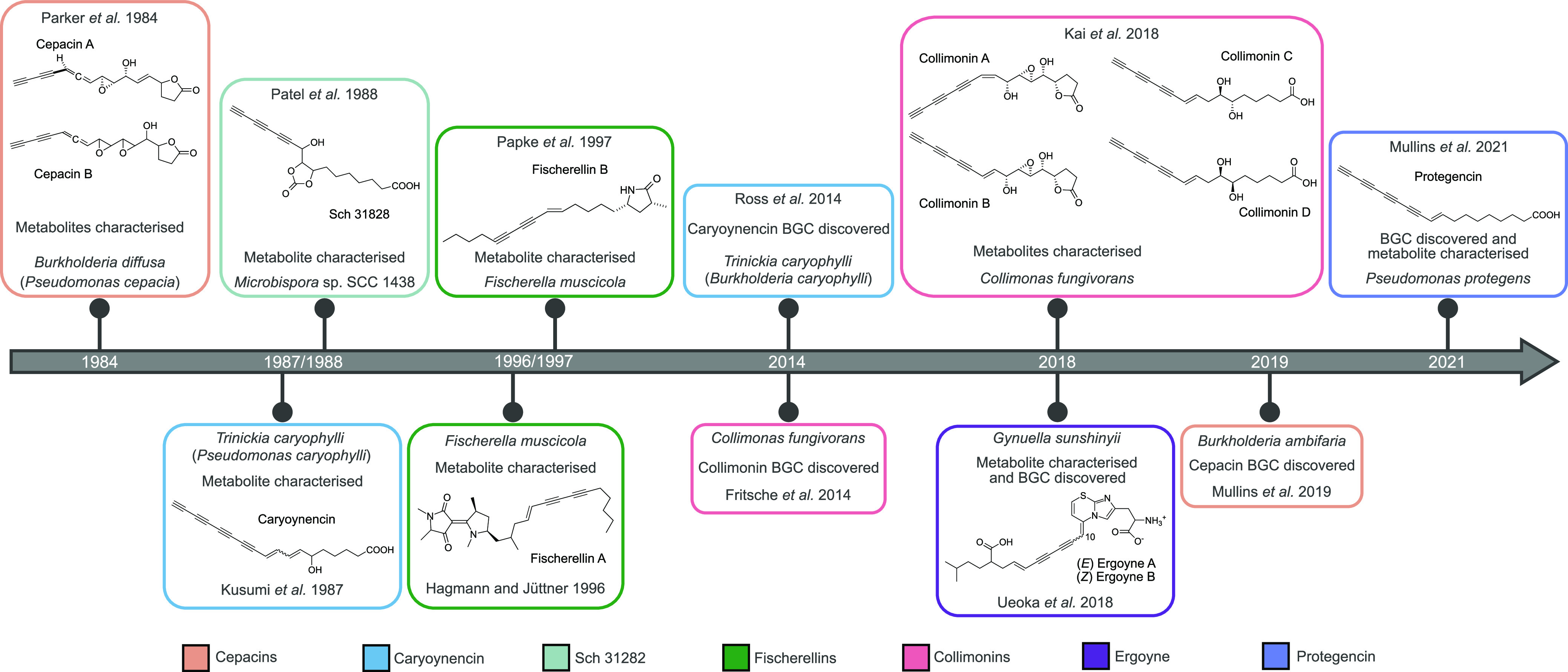
Timeline highlighting the discovery of polyyne metabolites and their biosynthetic gene clusters. The history of seven polyynes is displayed, highlighting the interval between the discovery of the metabolites and their BGCs.

Bacteria and fungi are an unparalleled source of structurally and functionally diverse metabolites with important applications in medicine and agriculture. Different classes of natural products can possess common structural features. One such moiety is the carbon-carbon triple (alkyne) bond. More than 65 alkyne-containing natural products have been isolated from marine bacteria and possess biotechnologically exploitable spectra of biological activity (1). Other metabolites possess elongated chains of alternating carbon-carbon single and triple bonds (polyynes). Polyynes have been isolated from diverse sources, including plants, fungi, bacteria, and even insects (2). The first bacterial polyynes, cepacins A and B, were discovered from the bacterium Burkholderia diffusa (formerly Pseudomonas cepacia) (3). However, the biosynthetic origin of the cepacins was only defined recently in the closely related species Burkholderia ambifaria, where these metabolites were shown to function in the biocontrol of damping off disease caused by the oomycete Globisporangium ultimum (4). The timeline of bacterial polyyne discovery is interesting, with multiple studies characterizing molecular diversity and different ecological roles (Fig. 1). Following the discovery of cepacins A and B in 1984 (3), several other polyynes were identified in *Proteobacteria*. Caryoynencin was isolated from Trinickia caryophylli (formerly Burkholderia caryophylli) (5) and Burkholderia gladioli (6). Alongside other antifungal compounds biosynthesized by *B. gladioli*, Lagriinae beetles exploit caryoynencin in a symbiotic relationship to protect their eggs from fungal attack (7). Collimonins were discovered from Collimonas fungivorans and displayed antifungal activity (8, 9), and ergoynes were found in the marine grass endophyte Gynuella sunshinyii (10) (Fig. 1). For the polyyne Sch 31828, isolated from *Actinobacteria* (11), and fischerellins A and B, isolated from *Cyanobacteria* (12, 13), the associated biosynthetic gene clusters (BGCs) remain unknown. While alkyne (14) and polyyne (6) biosynthetic mechanisms have been investigated, the evolution of polyyne biosynthesis, its relationship to alkyne biosynthesis, and overall polyyne diversity have yet to be established.

The influx of bacterial genomic assemblies over the last decade has revolutionized our understanding of bacterial evolution and enhanced our ability to discover natural products through multiple genome mining techniques (15). Common approaches for identifying the metabolic products of novel BGCs discovered by genome mining include comparative metabolic profiling following mutagenesis of target BGCs, activation/inactivation of cluster-situated regulators, and heterologous expression (15, 16). Alternative methods fueled by the increasing availability of genomic data include analyzing the evolutionary diversity of bacteria to identify lineages talented in specialized metabolite biosynthesis (15). A second, phylogeny-based mining strategy exploits the diversity of biosynthetic genes to discover natural product derivatives of known metabolites (15). Such an approach has the advantage of gleaning insight into the horizontal transfer of genes from BGCs by comparing biosynthetic gene trees to evolutionary phylogenies.

Considering the limited insights into polyyne evolution despite evidence of an evolutionarily broad distribution (4, 11) (Fig. 1), we sought to integrate existing knowledge and expand our understanding of the distribution of these structurally intriguing moieties. Here, we show their evolutionary history, by examining the co-occurrence of alkyne and polyyne biosynthetic cassettes (a minimum gene collection to biosynthesize a specific structural moiety), and their distribution, through a phylogeny-guided genome mining approach. This approach involved constructing a phylogeny based on genes associated with the alkyne and polyyne cassettes, identifying phylogenetic clades of interest, and mining representative genomes from these clades for uncharacterized polyyne biosynthetic gene clusters. Mixed evolutionary lineages within the alkyne phylogeny provided further evidence of their highly promiscuous nature. A distinct, monophyletic clade composed of polyyne biosynthetic gene clusters was observed within the broader alkyne gene cassette distribution. By examining subclade architecture, we identified a previously unexplored Pseudomonas polyyne clade that resulted in the characterization of a novel polyyne BGC, *pgn*, and its associated metabolite, protegencin.

## RESULTS

### Distribution of alkyne biosynthesis and emergence of polyyne biosynthesis.

**FIG 2:**
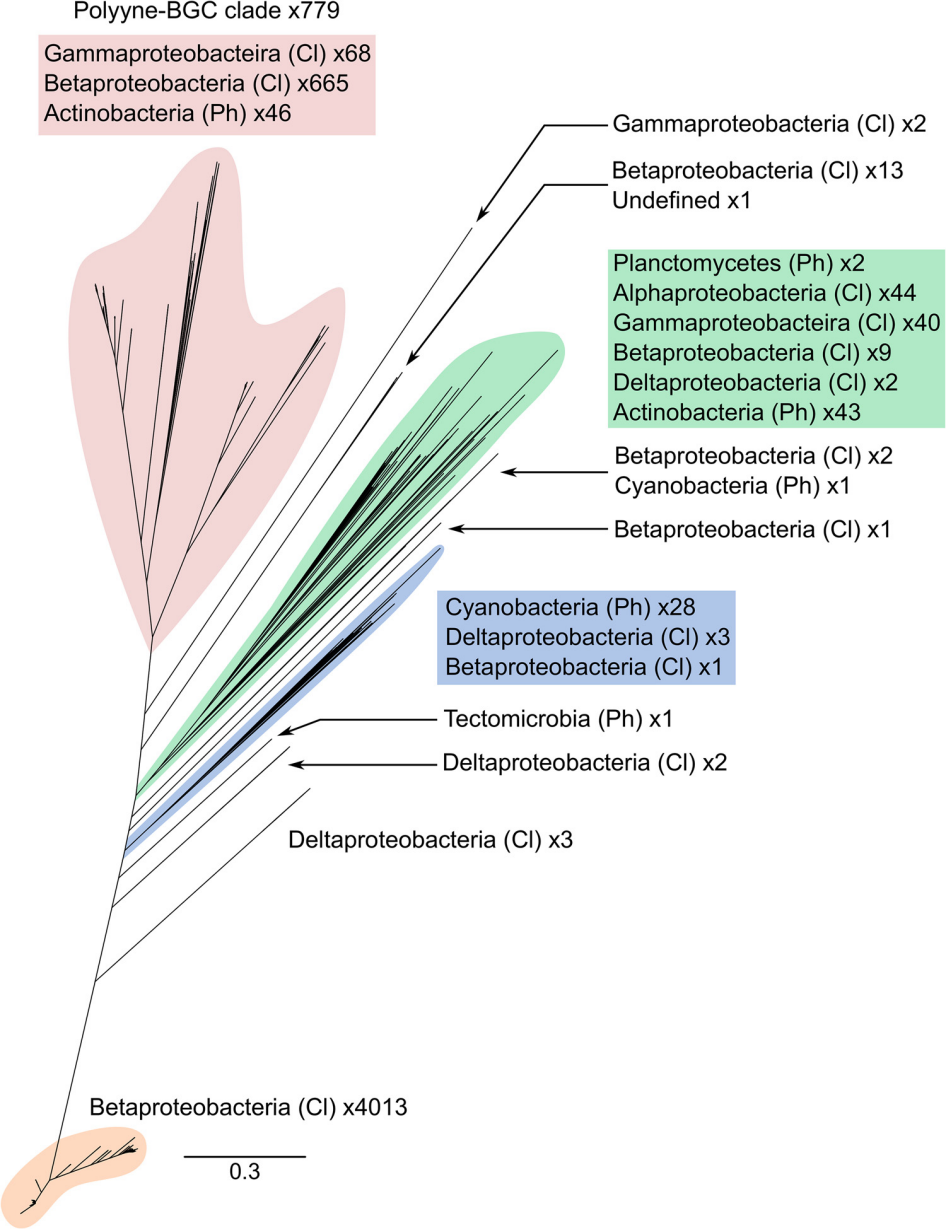
Fatty acyl-AMP ligase protein (JamA) phylogeny of potential alkyne-synthesizing bacteria. The phylogeny was constructed with 4,990 sequences using FastTree. The composition of each clade is indicated along with the number of representatives. Ph, phylum; Cl, class. Clades that contain multiple classes and phyla are highlighted with different colors.

A phylogenetic tree based on 4,990 protein sequences of the alkyne biosynthetic fatty acyl-AMP ligase, JamA, was constructed to assess the distribution of alkyne biosynthesis in bacteria (Fig. 2). Phylogenies were also constructed based on the corresponding gene, *jamA*, alongside the protein and gene sequences of the alkyne fatty acid desaturase JamB/*jamB*, and acyl carrier protein JamC/*jamC* (see Fig. S1 in the supplemental material).

10.1128/mBio.00715-21.2 FIG S1 Protein- and gene-based phylogenies of potential alkyne-biosynthesizing bacteria. Phylogenies were constructed based on 4,990 sequences of (a) fatty acyl-AMP ligase JamA, (b) desaturase JamB, and (c) acyl carrier protein JamC homologues, alongside their gene counterparts: (d) *jamA*, (e) *jamB*, and (f) *jamC*. The basal alkyne clade comprised of *Burkholderia* spp. is highlighted in blue, polyyne producers are highlighted in red, and the remaining deep-branching alkyne producers are highlighted in green. The general tree topology of the highlighted features is maintained across protein-based phylogenies, while the specific branching positions of subclades vary between phylogenies. Download FIG S1, PDF file, 0.2 MB. Copyright © 2021 Mullins et al. 2021 Mullins et al. https://creativecommons.org/licenses/by/4.0/ This content is distributed under the terms of the Creative Commons Attribution 4.0 International license.

The ability to biosynthesize alkynes was widely distributed across *Proteobacteria*, occurring in the *Alpha*-, *Beta*- *Delta*-, and *Gammaproteobacteria*, and represented 95.5% of available sequences (4,868 of 4,990). Within the *Proteobacteria*, *Betaproteobacteria* were the most dominant representatives at 96.6% (4,704 of 4,868 *Proteobacteria*) and occurred in multiple deep-branching lineages, potentially indicating several acquisition events into the phylum (Fig. 2), which is also supported by the additional phylogenies of alkyne biosynthetic genes and proteins (Fig. S1). However, the rearrangement of the branchpoints observed in the JamABC/*jamABC* protein and gene phylogenies confounds the ability to determine the number of horizontal gene transfer events that have occurred (Fig. S1). Despite these phylogenetic limitations, all six phylogenies (Fig. 2; Fig. S1) supported a similar overarching topology. Most sequences (80% [4,013 of 4,990]) occurred in a basal clade composed entirely of *Burkholderia* species, including B. pseudomallei, B. thailandensis, and *B. ubonensis* (Fig. 2), while the opposing end of the unrooted phylogeny consistently encompassed 779 sequences with a congruent topology (Fig. 2; Fig. S1). Outside of the *Proteobacteria*, examples of the alkyne cassette were found in members of the *Cyanobacteria* (29 genomes), *Planctomycetes* (2 genomes), and the candidate phylum *Tectomicrobia* uncultivated sponge symbiont “Candidatus Entotheonella” (1 genome).

Construction of the phylogeny of the biosynthetic fatty acyl-AMP ligase JamA also highlighted a discrepancy in the literature regarding the previously characterized B. pseudomallei alkyne biosynthetic locus (14). Inclusion of the purported JamA homologue alongside the JamA homologue identified during this analysis confirmed the latter to be the genuine JamA homologue (see Fig. S2 in the supplemental material). Annotation of the biosynthetic locus revealed the genuine fatty acyl-AMP ligase was encoded downstream of the previously characterized JamA protein (Fig. S2).

10.1128/mBio.00715-21.3 FIG S2 Fatty acyl-AMP ligase protein-based phylogenies. Comparison of the tree topologies of the JamA phylogeny with the presence and absence of the FAAL proteins identified in the B. pseudomallei alkyne biosynthesis locus. Key clades are indicated by color to aid interpretation of tree topologies. (a) Alkyne biosynthetic locus of B. pseudomallei K96243 with homologues of *jamABC* highlighted. (b) Phylogeny of JamA homologues identified in this study (also displayed in Fig. 2). (c) Replacement of the B. pseudomallei JamA homologue identified in this study with the FAAL protein identified by Zhu et al. (14). (d) JamA phylogeny with B. pseudomallei FAAL proteins from this study and that of Zhu et al. (14) included. Download FIG S2, PDF file, 0.1 MB. Copyright © 2021 Mullins et al. 2021 Mullins et al. https://creativecommons.org/licenses/by/4.0/ This content is distributed under the terms of the Creative Commons Attribution 4.0 International license.

**FIG 3:**
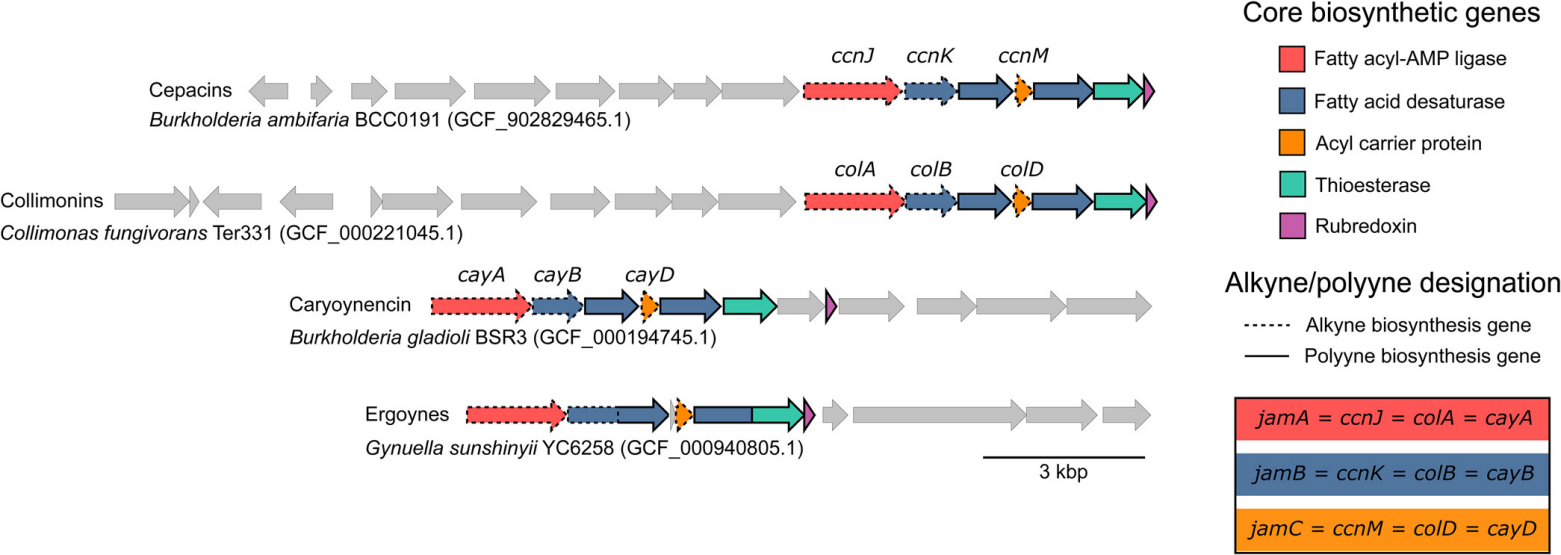
Comparison of gene organization between characterized polyyne biosynthetic gene clusters. Genes associated with alkyne biosynthesis are indicated by a bold outline: fatty acyl-AMP ligase, *jamA*; desaturase, *jamB*; and the acyl carrier protein, *jamC*. Genes identified as polyyne biosynthesis-specific genes are indicated by a dashed outline: two further desaturase genes, a thioesterase gene, and a rubredoxin gene. Biosynthetic gene cluster (BGC)-specific nomenclature for *jamABC* homologues is included for cepacin, caryoynencin, and collimonin BGCs. The gene nomenclature of the ergoyne BGC is unavailable. The NCBI locus tags for the polyyne biosynthetic gene clusters in the representative RefSeq genomes are as follows: *B. ambifaria* BCC0191 (GCF_902829465.1), HWW27_RS03890 to HWW27_RS03965; *C. fungivorans* Ter331 (GFA_000221045.1), CFU_RS05585 to CFU_RS05660; *B. gladioli* BSR3 (GCF_000194745.1), BGLA_RS09975 to BGLA_RS10025; and *G. sunshinyii* YC6258 (GCF_000940805.1), YC6258_RS21350 to YC6258_RS27625.

To understand the broader relationship between bacterial alkyne and polyyne biosynthesis, a comparison of characterized polyyne biosynthetic gene clusters was performed. Analysis of the gene content and architecture of four characterized/published polyyne BGCs (for cepacins, collimonins, caryoynencin, and ergoynes) identified seven common genes (Fig. 3). In addition to the three genes encoding the alkyne biosynthetic cassette, *jamABC* (14), genes encoding two additional fatty acid desaturases, a thioesterase, and rubredoxin were found in all BGCs (Fig. 3). Using this knowledge, we screened DNA sequences flanking the *jamABC* alkyne biosynthetic cassettes for the presence of the remaining four genes. This revealed a monophyletic clade in the alkyne phylogenies (Fig. 2; Fig. S1) where the 779 corresponding genomes possessed the conserved polyyne gene cassette (Fig. 3), with a few exceptions. Three discrepancies were observed within the monophyletic polyyne clade: *B. gladioli* strain 3848s-5 and three *Streptomyces* strains appeared to lack the colocalized thioesterase and rubredoxin genes with the remaining polyyne core biosynthetic genes, but manual inspection of these genomes revealed the BGCs were split across two contigs. A subset of 10 actinobacterial genomes appeared to have the thioesterase- and rubredoxin-encoding genes replaced by a gene encoding a cytochrome P450. These 10 genomes represented three genera (*Streptomyces*, *Micromonospora*, and *Amycolatopsis*) and were confined to a single subclade in the monophyletic polyyne clade. The final discrepancy included two representatives of the family *Mycobacteriaceae* that lacked the rubredoxin gene.

**FIG 4:**
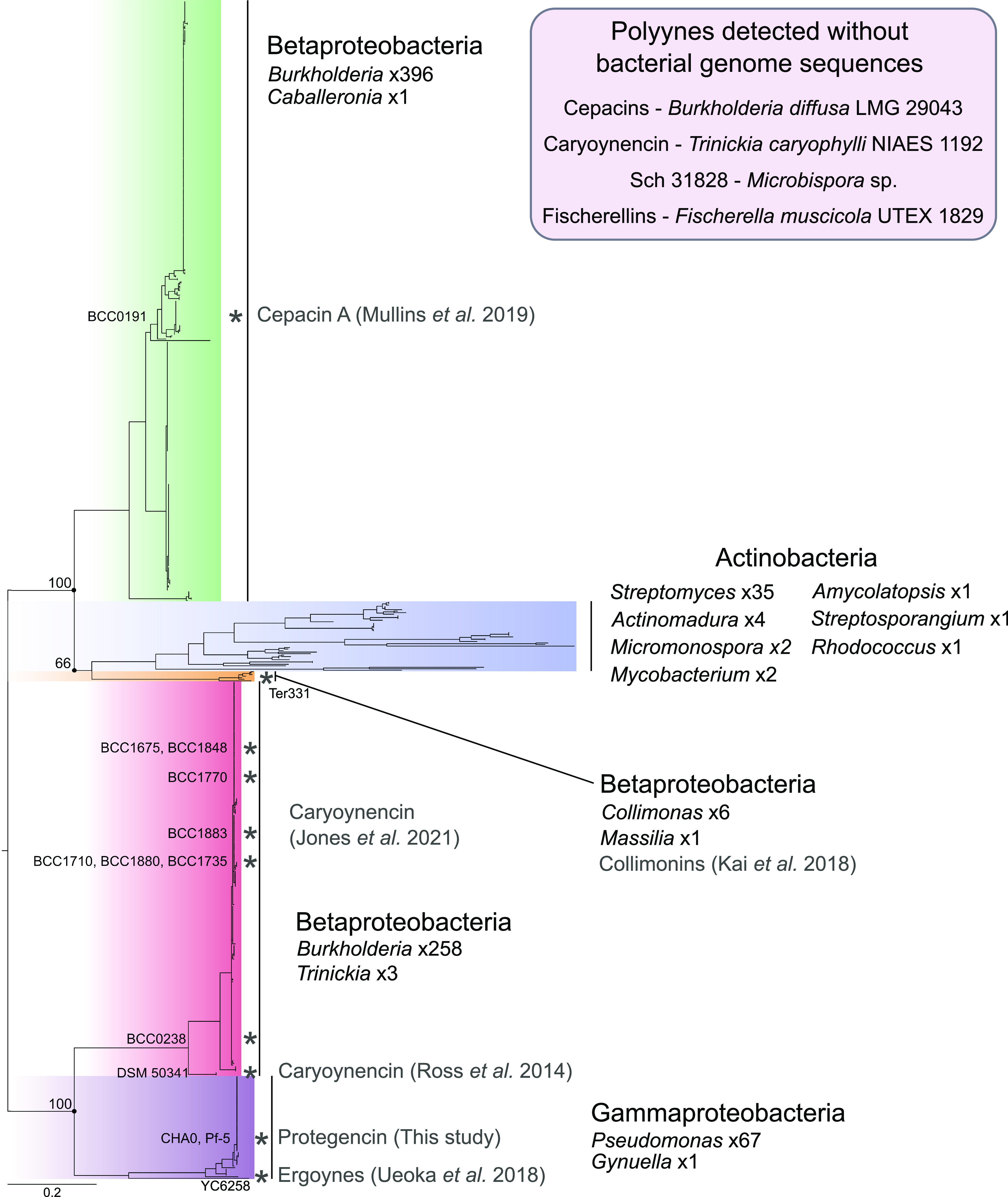
Desaturase protein-based phylogeny of polyyne-producing bacteria. Homologues of the cepacin desaturase CnnN (protegencin PgnH) were extracted from bacterial genomes represented in the monophyletic alkyne clade as polyyne producers. The four *Proteobacteria* clades, their composite genera, and associated polyyne metabolites are indicated, in addition to the *Actinobacteria* phylum clade. The *Gammaproteobacteria* clade was used as the root based on the topologies of alkyne gene phylogenies. Known polyyne producers are indicated with asterisks, and the specific strains are labeled. Bootstrap values are indicated for splits between the 5 major clades. The scale bar represents the number of substitutions per position.

To investigate the diversity of the monophyletic clade, a separate phylogeny was constructed based on one of the polyyne-associated desaturase proteins (Fig. 4). This phylogeny was rooted using the basal branches of the clade of interest from both the JamA and JamB phylogenies (Fig. 2): a *Gammaproteobacteria* subclade and *Betaproteobacteria* subclade. Within the resulting phylogeny, we defined five major clades representing three *Betaproteobacteria* clades, one *Gammaproteobacteria* clade, and an *Actinobacteria* clade (Fig. 4). Each of the four previously characterized polyynes corresponded to a different clade, with collimonins, caryoynencin, and cepacins localized to the three distinct *Betaproteobacteria* clades (Fig. 4). The ergoynes, biosynthesized by *G. sunshinyii*, were in the *Gammaproteobacteria* clade, but with deep branching separating *G. sunshinyii* from the remainder of the clade members (Fig. 4). Each *Proteobacteria* clade was dominated by a single genus and mainly structured with relatively shallow branching. In comparison, the *Actinobacteria* clade possessed deep branching and contained representatives of seven genera, including *Micromonospora*, *Actinomadura*, and *Rhodococcus*, but was dominated by *Streptomyces* species. This analysis identified the cepacin BGC in several species that were previously not known to carry the gene cluster (Fig. 4), including *B. contaminans*, *B. vietnamiensis* and Caballeronia peredens.

### Exploration of the *Gammaproteobacteria* clade reveals an uncharacterized polyyne.

**FIG 5:**
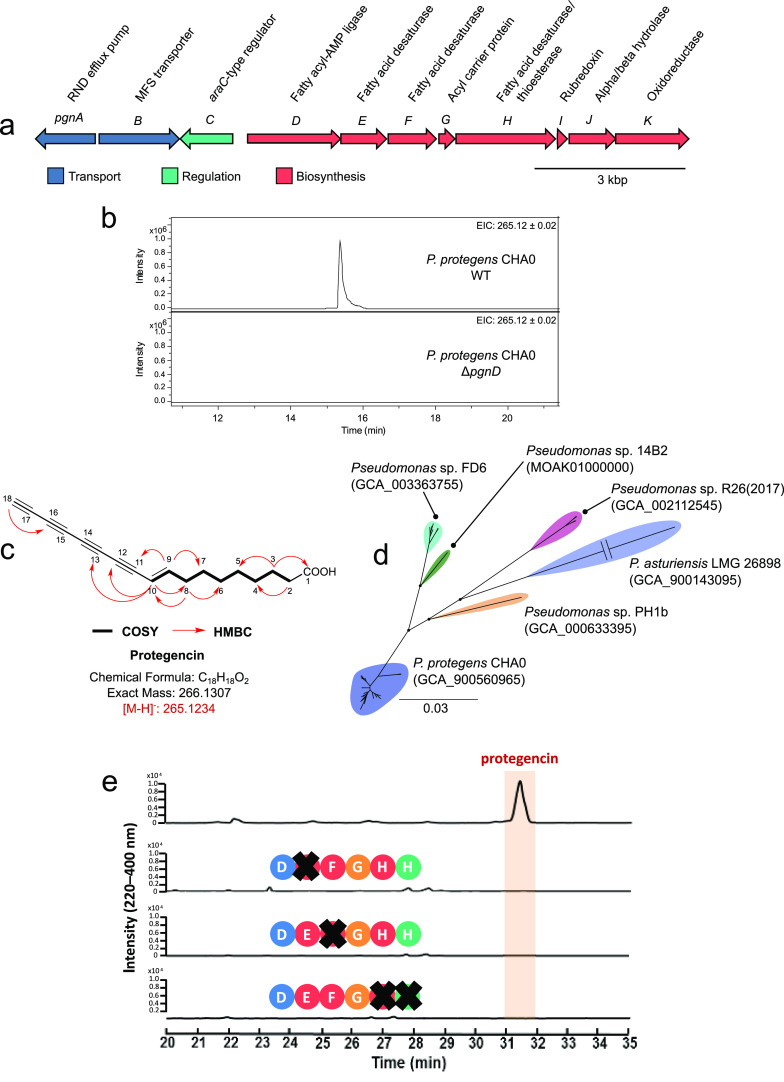
Organization and distribution of the protegencin (*pgn*) BGC and analysis of protegencin production. (a) Organization and putative function of genes within the *pgn* BGC. (b) Extracted-ion chromatograms at *m/z *= 265.12 ± 0.02, corresponding to [M − H]^−^ for protegencin, from LC-MS analyses of crude extracts made from agar-grown cultures of P. protegens CHA0 (top) and the P. protegens CHA0 Δ*pgnD* mutant (bottom). (c) Structure of protegencin, determined by a combination of high-resolution mass spectrometry and NMR spectroscopy (see Table S2 and Fig. S3a to g). (d) Core gene-based phylogeny, using 1,487 genes, of 67 Pseudomonas genomes carrying the *pgn* BGC. The main nodes that demarcate the Pseudomonas species are highlighted, and all possess bootstrap values of 100. Representative strains and genome assembly accession numbers are included for each defined species. The scale bar represents the number of substitutions per site. The *P. asturiensis* branch was shortened (indicated by a break), and as such, the scale bar does not apply. (e) HPLC chromatograms (220 to 400 nm) of P. protegens Pf-5 wild-type and in-frame insertional mutant cultures. Only in the presence of all three desaturase genes (*pgnE*, *pgnF*, and *pgnH*) is protegencin produced. No polyyne precursors can be detected in the mutant strains.

Aside from the single representative of the *Gynuella* genus, the *Gammaproteobacteria* clade was dominated by Pseudomonas. However, this genus is not known to produce polyynes. Evidence of a Pseudomonas polyyne BGC has been alluded to as a homologous gene cluster of the collimonin (8) and caryoynencin (6) BGCs during the discovery of these polyynes. As such, we sought to investigate the production of an uncharacterized polyyne in Pseudomonas (Fig. 5a), focusing on Pseudomonas protegens (formerly P. fluorescens) strains Pf-5 and CHA0 as model systems (see Table S1 in the supplemental material). High-performance liquid chromatography (HPLC) analysis of these two strains revealed a small chromatographic peak with a characteristic UV absorbance spectrum as observed for other polyynes (6, 8). Comparative negative-ion-mode high-resolution electrospray ionization quadrupole time of flight mass spectrometry (HR-ESI-Q-TOF MS) analysis of the wild-type P. protegens Pf-5 and CHA0 strains and mutants with in-frame deletions in the fatty acyl-AMP ligase gene (Pf-5 Δ*pgnD* and CHA0 Δ*pgnD*, respectively) identified a compound, which we named protegencin, with the molecular formula C_18_H_18_O_2_ (Calculated for C_18_H_17_O_2_^−^: 265.1234. Found: 265.1239) as the product of the polyyne BGC (Fig. 5b and c; see Fig. S3a and b in the supplemental material).

10.1128/mBio.00715-21.4 FIG S3 (a) High-resolution mass spectrometry analysis of P. protegens CHA0 producing protegencin. Measured spectrum of protegencin gives [M − H]^−^, [(M − 2H) − Na]^−^ and [(M − CO_2_) −H]^−^ ions. The generated molecular formulas for each species are shown and are in agreement with the molecular formula of protegencin. (b) LC-MS analysis of protegencin production by P. protegens Pf-5. Extracted-ion chromatograms at *m/z* = 265.12 ± 0.02, corresponding to [M − H]^−^ for protegencin, from LC-MS analyses of crude extracts made from agar-grown cultures of P. protegens Pf-5 WT (top) and the Δ*pgnD* mutant (bottom). (c) ^1^H NMR spectrum of protegencin in DMSO-*d*_6_ at 500 MHz. (d) ^13^C NMR spectrum of protegencin in DMSO-*d*_6_ at 125 MHz. (e) HSQC NMR spectrum of protegencin in DMSO-*d*_6_ at 500 MHz. (f) COSY NMR spectrum of protegencin in DMSO-*d*_6_ at 500 MHz. (g) HMBC NMR spectrum of protegencin in DMSO-*d*_6_ at 500 MHz. Download FIG S3, PDF file, 0.5 MB. Copyright © 2021 Mullins et al. 2021 Mullins et al. https://creativecommons.org/licenses/by/4.0/ This content is distributed under the terms of the Creative Commons Attribution 4.0 International license.

10.1128/mBio.00715-21.6 TABLE S1 Strains and plasmids used in this study for mutagenesis. Download Table S1, PDF file, 0.1 MB. Copyright © 2021 Mullins et al. 2021 Mullins et al. https://creativecommons.org/licenses/by/4.0/ This content is distributed under the terms of the Creative Commons Attribution 4.0 International license.

10.1128/mBio.00715-21.7 TABLE S2 ^1^H (500 MHz) and ^13^C (125 MHz) NMR spectroscopic data of protegencin. Download Table S2, PDF file, 0.1 MB. Copyright © 2021 Mullins et al. 2021 Mullins et al. https://creativecommons.org/licenses/by/4.0/ This content is distributed under the terms of the Creative Commons Attribution 4.0 International license.

### NMR spectroscopy confirms protegencin is a novel Pseudomonas polyyne.

Polyynes are notorious for being unstable and difficult to isolate, with recent studies requiring derivatization by click chemistry prior to spectroscopic analysis (6). The isolation of protegencin required careful optimization to enable spectroscopic characterization of the compound without derivatization. Purified fractions of protegencin were dried under vacuum for 2 to 3 h, with the addition of small volumes of MeCN to promote the removal of water from the sample. Freeze-drying of protegencin-containing fractions resulted in a polymerized brown oil. Using this procedure, protegencin was isolated as a brownish, amorphous powder. Its ^1^H, ^13^C, correlation spectroscopy (COSY), heteronuclear single quantum coherence (HSQC), and heteronuclear multiple-bond correlation (HMBC) spectra were acquired in deuterated dimethyl sulfoxide (DMSO-*d*_6_) (see Table S2 and Fig. S3c to g in the supplemental material). The ^1^H NMR spectroscopic data displayed two olefinic protons (δ_H_ 6.65, 1H, dt, *J *= 16.0, 6.5, H-9; δ_H_ 5.79, 1H, d, *J *= 16.0 H-10), a methine proton (δ_H_ 4.06, 1H, H-18), and seven pairs of methylene protons. The ^13^C NMR and HSQC spectroscopic data (Table S2) indicated 18 carbons, including three methine carbons (δ_C_ 155.4, 107.3, and 74.7), seven methylene carbons (δ_C_ 34.1, 33.4, 28.9 × 2, 28.8, 28.0, and 24.9), one carbonyl carbon (δ_C_ 175.0.), and seven quaternary carbons. The above data suggested a similar polyyne structure to caryoynencin (5, 6), but lacking a pair of olefinic protons and an oxymethine proton. The structure was further established by COSY and HMBC spectroscopic data analysis (Fig. S3f and g). The HMBC correlations of H-9 with C-11, C-8, and C-7, along with the couplings of H-10 to C-9, C-11, C-12, C-8, and C-13, confirmed a double bond was located at C-9/C-10 next to the polyyne scaffold, as observed in caryoynencin. The double bond at C-7/C-8 and hydroxyl group at C-6 in caryoynencin were missing in protengencin, as evidenced by HMBC correlations from a methylene (H_2_-8) to two methine carbons (C-9 and C-10) and two methylene carbons (C-6 and C-7), and from a methylene (H_2_-4) to two methylene carbons (C-6 and C-5), as well as COSY couplings of H_2_-8 to H-9 and H_2_-7. The other COSY correlations of H_2_-3 to H_2_-4 and H_2_-2, and of H_2_-4 to H_2_-5, together with HMBC correlations of H-2 to C-1, C-3, and C-4, and of H-3 with C-1, C-2, C-4, and C-5, confirmed the structure of the saturated region of this metabolite. Therefore, the structure of protegencin was elucidated as a novel polyyne natural product, as shown in Fig. 5c.

### Distribution of protegencin (*pgn*) BGC within Pseudomonas.

Following the discovery of the previously uncharacterized polyyne metabolite protegencin, we sought to fully understand the species distribution of the *pgn* locus. The Pseudomonas branches of the *Gammaproteobacteria* clade represented 67 Pseudomonas genomes. Subsequent average nucleotide identity analysis (ANI) of these genomes indicated the presence of multiple species. Based on the established 95% species delineation threshold for ANI (17, 18), six species were identified: these included two named species, Pseudomonas protegens (P. fluorescens group) and Pseudomonas asturiensis (P. syringae group) (19), and four unnamed species. The relatedness of these two species to one another is highlighted in the core-gene-based phylogeny (Fig. 5d). P. protegens was the dominant species possessing the *pgn* BGC, representing approximately 75% of genomes. A wider search for genome representatives of these six species in the European Nucleotide Archive (ENA) revealed that all genomes available of these species possess the protegencin (*pgn*) BGC, except for *P. asturiensis*. Of the two available *P. asturiensis* genomes, only the type strain LMG 26898^T^ contained the *pgn* BGC. It was absent from Pseudomonas sp. strain 286 (98.9% ANI to LMG 26898). The *pgn* locus is present in five out of six Pseudomonas species examined in this study.

### A conserved desaturase triad is essential for polyyne formation.

The high conservation of the three desaturase genes and the thioesterase gene across all orthologous polyyne BGCs is notable (Fig. 3). To elucidate their roles, we performed targeted gene replacements. Specifically, we individually replaced the desaturase and thioesterase genes with a kanamycin and apramycin resistance cassette in the P. protegens *pgn* and *T. caryophylli cay* BGCs, respectively (Fig. 5e; see Fig. S4 in the supplemental material). Sequence analyses indicated that pairs of desaturase genes (*pgnE*/*cayB* and *pgnF*/*cayC*) would have similar functions. The deduced product of *pgnH* is a didomain enzyme with putative desaturase and thioesterase functions that corresponds to *cayE* and *cayF*, respectively. The metabolic profiles of the mutant strains were compared by HPLC (220 to 400 nm) with those of the wild-type strains, with or without the empty pGL42a or pJET1.2/blunt vector used for mutagenesis (Fig. 5e; Fig. S4). Whereas P. protegens Pf-5 (with or without the empty vector) produces protegencin, in the Δ*pgnE* Kan^r^, Δ*pgnF* Kan^r^, and Δ*pgnH* Kan^r^ mutant strains, no polyyne precursor could be identified (Fig. 5e). Deletions of the desaturase genes *cayB*, *cayC*, and *cayE* and the thioesterase gene *cayF* in *T. caryophylli* abolished the production of caryoynencin. The wild type (with or without an empty vector) generates the 7*E*/*Z*-isomers of caryoynencin, but the mutant strains (Δ*cayB* Apr^r^, Δ*cayC* Apr^r^, Δ*cayE* Apr^r^, and Δ*cayF* Apr^r^) produce neither polyynes nor pathway intermediates (Fig. S4). These data indicate that the three desaturases and the thioesterase synergize in the production of polyynes. Interestingly, the same multienzyme system that gives rise to a tetrayne in the protegencin and caryoynencin BGCs appears to form a triyne in the collimonin pathway and a diynyl allene in the cepacin pathway (Fig. 1).

10.1128/mBio.00715-21.5 FIG S4 HPLC analyses (220 to 400 nm) of the metabolite extracts from the *cay* mutants of *T. caryophylli.* Shown are HPLC profiles of the *T. caryophylli* wild-type strain and mutants deficient in desaturase (*cayB*, *cayC*, and *cayE*) and thioesterase (*cayF*) genes. Caryoynencin can only be detected in the wild-type culture. The mutant strain cultures (Δ*cayB*, Δ*cayC*, Δ*cayE*, and Δ*cayF*) do not produce any detectable polyyne precursors. Download FIG S4, PDF file, 0.1 MB. Copyright © 2021 Mullins et al. 2021 Mullins et al. https://creativecommons.org/licenses/by/4.0/ This content is distributed under the terms of the Creative Commons Attribution 4.0 International license.

## DISCUSSION

### Highly transmissible alkyne and polyyne cassettes.

Our results identify evidence of a single point of evolution of polyyne biosynthesis within bacteria and demarcate its evolution from alkyne biosynthesis (Fig. 2). The basal positioning of *Proteobacteria* within the polyyne phylogeny hints at a potential origin of this biosynthetic ability (Fig. 4), followed by horizontal gene transfer into *Actinobacteria* and other *Proteobacteria* classes. Additionally, the occurrence of alkyne biosynthetic genes across diverse bacterial lineages was also indicative of multiple horizontal gene transfer events. Few other fatty acid synthase-based biosynthetic capabilities appear to occur across a spectrum of bacterial lineages.

While examples of polyyne biosynthesis exist across plants, fungi, and insects, they appear to have different biosynthetic origins compared to bacteria (2). In contrast to the biosynthetic mechanism for multiple carbon-carbon triple bond formation defined in this study, there is no evidence of other biosynthetic pathways evolving from an alkyne precursor biosynthetic gene cassette. Within bacteria, a separate, evolutionarily independent, mechanism exists for the biosynthesis of multiple carbon-carbon triple bonds in the form of enediynes (20). In contrast to the seven-gene cassette required for polyyne biosynthesis, a minimal five-gene cassette was defined by comparing 10 biosynthetic pathways associated with production of enediyne-containing natural products (20). Mining of bacterial genomes revealed comparably fewer examples of the enediyne gene cassette (20, 21); however, there is evidence of horizontal gene transfer across several phyla (20) similar to the alkyne and polyyne gene cassettes.

### Phylogeny-driven metabolite discovery.

Mapping the diversity of polyyne biosynthetic gene clusters through functional gene and protein phylogenies permitted the discovery of an uncharacterized Pseudomonas polyyne BGC, *pgn*, and metabolite, protegencin. Hotter et al. (22) have recently demonstrated that this P. protegens polyyne, protegencin, acts as an algicidal toxin of the green alga Chlamydomonas reinhardtii. In parallel to these studies characterizing protegencin, Murata et al. (23) identified the same polyyne biosynthetic gene cluster in the biocontrol strain P. protegens Cab57, designating the molecules produced as protegenins.

Function-based phylogenies have been exploited previously to gain insight into natural product diversity. For example, ketosynthase (KS) and condensation (C) domains have been used to identify polyketide synthase (PKS) and nonribosomal peptide synthetase (NRPS) BGCs, respectively (24). Mining for genes known to encode enzymes that biosynthesize specific structural moieties also enables discovery and comparison to other structurally related metabolites. A novel glutarimide, gladiostatin, was recently discovered in Burkholderia gladioli by identifying a BGC possessing genes similar to those associated with the biosynthesis of glutarimide antibiotics in *Streptomyces* species (25, 26).

The deep branching observed within the *Actinobacteria* clade of the polyyne phylogeny represents evidence of sequence divergence and may translate into structural diversity of the resulting polyyne natural products. No *Actinobacteria* polyyne has been associated with a biosynthetic gene cluster to date, and the only published *Actinobacteria* polyyne, Sch 31828, originated from a strain that lacks a genome sequence and has not been characterized at the species level, *Microbispora* sp. strain SCC 1438 (11). In *Cyanobacteria*, many alkyne-containing natural products have been characterized (1); in contrast, only two polyynes have been discovered to date (12, 13). The lack of a genome sequence for the Fischerella muscicola strains that produce fischerellins also impedes our mapping of their phylogenetic relationship to other polyyne biosynthetic gene clusters, and they potentially represent an uncharacterized *Cyanobacteria* clade.

### Evidence for an uncharacterized polyyne in P. protegens.

We identified and characterized a novel Pseudomonas polyyne metabolite produced by the widely studied P. protegens strains Pf-5 and CHA0 (Table S1). Both strains have an extensive history of biopesticidal properties (27, 28), indicative of the array of potent antimicrobial natural products biosynthesized by this species, such as the antifungal metabolites 2,4-diacetylphloroglucinol and pyoluteorin (27, 28). Previous sequence comparisons had highlighted the existence of a polyyne BGC in P. protegens with similarities to the caryoynencin (6) and collimonin (8) BGCs. However, homology to only the core biosynthetic region was defined in these studies (6, 8) (Fig. 2), and the metabolic product was not identified. Additionally, a transcriptomics analysis of the Gac global regulatory system highlighted a locus possessing similarities to those in *Burkholderia* (29), with a gene organization and putative gene functions like those found in the cepacin BGC (4).

Overall, we sought to understand the evolution and diversity of polyyne biosynthesis following emergence from the alkyne biosynthetic gene cassette. This study exploited functional gene phylogenetics alongside evolutionary analyses to explore polyyne biosynthetic diversity. Bioinformatics analyses supported by molecular biology and analytical chemistry led to the discovery of a Pseudomonas-derived polyyne BGC, *pgn*, and its metabolic product, protegencin. The conserved multienzyme system was proven to be essential for polyyne formation in both protegencin and caryoynencin biosynthesis (Fig. 5e). Discovering novel polyynes and investigating their biosynthetic mechanisms will support future endeavors to better understand these unusual biologically active metabolites.

## MATERIALS AND METHODS

### Detection of alkyne and polyyne biosynthetic gene clusters.

A BLASTp (30) search of NCBI genomes, excluding *Burkholderia* (taxid: 32008) and a local database of *Burkholderia* assemblies (3,002 downloaded genomes and 4,434 genomes assembled from publicly available Illumina read data) was performed with the cepacin homologue (CcnK) (4) of the desaturase JamB as the query. *Burkholderia* genomic assemblies were downloaded from the European Nucleotide Archive (ENA) using a script from enaBrowserTools (https://github.com/enasequence/enaBrowserTools). The local assemblies constructed from publicly available Illumina paired-end fastq data were assembled via Shovill v0.9.0 (https://github.com/tseemann/shovill). The top 5,000 genus and species hits from NCBI were dereplicated, and their associated genomes were downloaded and combined with the local collection. The flanking 30-kbp sequence of the protein hit (E value of <1.00e−50) was extracted, and the encoded protein domains were predicted using Interproscan v5.38-76.0 (31). Each sequence was screened for the presence of three domains corresponding to the presence of a fatty acyl-AMP ligase (IPR040097), fatty acid desaturase (IPR005804), and acyl carrier protein (IPR009081). The presence of these three homologues was considered evidence of alkyne biosynthesis potential. These sequence fragments were further screened for the presence of four additional protein homologues—two desaturases, a thioesterase, and a rubredoxin protein—via BLASTp, to determine the potential of polyyne biosynthesis. A threshold of 1.00e−100 was used to determine the presence of the additional desaturase proteins based on a noticeable change in E value between protein presence and absence. Manual analysis of the sequence fragments for the presence or absence of alkyne- and polyyne-associated genes was necessary to define the thioesterase and rubredoxin thresholds due to an indistinct change in E value and BGCs occurring near contig edges.

### Phylogenetic and phylogenomic analyses of alkyne and polyyne BGCs.

Protein and nucleotide alignments were generated using MAFFT v7.455 (32), with the exception of core gene alignments, which were generated with Roary v3.13.0 (33). Alkyne-related phylogenies were constructed using multithreaded FastTree v2.1.10 with a general time-reversible model and gamma distribution for nucleotide alignments (34). The remaining phylogenies were constructed using RAxML v8.2.12 (35) with a general time-reversible model and gamma distribution supported by 100 bootstraps. In cases where the protein or gene sequence of interest occurred as a fusion, the region of interest was extracted for use in the alignment. Bacterial genomes were annotated with Prokka v1.14.5 (36). Average nucleotide identity (ANI) analyses were initially performed with fastANI v1.2 (17) and supported by PyANI v0.2.9 (mummer) (37). A comparison of the annotated sequences was visualized using Easyfig (38).

### Mutagenesis of polyyne biosynthetic gene clusters.

A range of in-frame, gene replacement, and insertional inactivation mutants were constructed in P. protegens and *T. caryophylli* (Table S1) to link polyyne biosynthesis to gene clusters and cassette function as described in the supplemental material.

### Metabolite extraction and LC-MS analysis of P. protegens wild types and *ΔpgnD* mutants.

P. protegens wild-type strains (CHA0 and Pf-5) and mutants (CHA0 Δ*pgnD* and Pf-5 Δ*pgnD*) were grown in LB broth at 30°C overnight with agitation and then inoculated onto pea exudate medium (PEM) agar plates (see supplemental material for PEM constituents). After incubation on PEM agar at 22°C for 3 days, the medium in a single plate was cut into approximately 1- by 1- by 0.5-cm pieces after removing surface growth and extracted with 10 ml of ethyl acetate (EtOAc), submerging the agar pieces, for 2 h static with periodic agitation. The crude extract was then filtered, followed by rotary evaporation and redissolving in 1 ml of 50% acetonitrile in water. The crude extracts were then analyzed by ultrahigh-performance (UHPLC)-ESI-Q-TOF MS after centrifugation to remove debris. UHPLC-ESI-Q-TOF MS analysis was performed using a Dionex UltiMate 3000 UHPLC device connected to a Zorbax Eclipse Plus C_18_ column (100 by 2.1 mm, 1.8 μm) coupled to a Bruker MaXis Impact mass spectrometer. The mobile phases consisted of water and acetonitrile (MeCN), each supplemented with 0.1% formic acid. After 5 min of isocratic elution at 5% MeCN, a gradient of 5 to 100% MeCN in 12 min was employed with a flow rate 0.2 ml min^−1^, followed by isocratic elution for a further 5 min and then returning to the initial conditions within 3 min. The mass spectrometer was operated in positive-ion or negative-ion mode with a scan range of 50 to 3,000 *m/z*. The source conditions were end-plate offset at −500 V, capillary at −4,500 V, nebulizer gas (N_2_) at 1.6 bars, dry gas (N_2_) at 81 min^−1^, and dry temperature at 180°C. The ion transfer conditions were ion funnel radio frequency (RF) at 200 Vpp, multiple RF at 200 Vpp, quadrupole low mass at 55 *m/z*, collision energy at 5.0 eV, collision RF at 600 Vpp, ion cooler RF at 50 to 350 Vpp, transfer time at 121 μs, and prepulse storage time at 1 μs. Calibration was performed with 1 mM sodium formate through a loop injection of 15 μl at the start of each run. Additional LC-MS methods are described in the supplemental material.

### Preparative HPLC purification and structure elucidation by NMR spectroscopy.

P. protegens Pf-5 metabolite production was scaled up by growth on 53 PEM agar plates (1.5 liters of medium in total). After growth at 22°C for 3 days, the medium was processed as described for the LC-MS analyses. The purification was performed on an Agilent 1200 series HPLC instrument equipped with a diode array detector and an Agilent Zorbax C_18_ column (100 by 21.1 mm, 5 μm), and the crude EtOAc extract was separated with an MeCN-H_2_O gradient (0 min, 5% MeCN; 5 min, 30% MeCN; 50 min, 30% MeCN; 80 min, 100% MeCN; 90 min, 100% MeCN) at a flow rate of 9 ml/min and monitoring absorbance at 260 nm. This resulted in the isolation of a putative polyyne metabolite (1.5 mg, *t*_R_ = 76.8 min). The structure of this compound was elucidated using NMR spectroscopy. The sample was dissolved in 0.6 ml of deuterated DMSO in a Norell standard series 5-mm NMR tube, and 1D/2D spectra (^1^H, ^13^C, COSY, HSQC, and HMBC) were obtained at 500 MHz for ^1^H NMR and 125 MHz for ^13^C NMR on a Bruker Avance III HD 500-MHz spectrometer. Chemical shifts (δ) are given in ppm, and coupling constants (*J*) are given in hertz (Hz). Additional HPLC methods are described in the supplemental material.

### Data availability.

All bacterial genome assemblies and Illumina reads analyzed during this study were downloaded from the National Center for Biotechnology Information (NCBI) or European Nucleotide Archive public databases.

10.1128/mBio.00715-21.1 TEXT S1 Detailed methods for the construction of gene insertion and gene replacement mutants in P. protegens and *T. caryophylli* and the method for preparing pea exudate medium for the induction of specializeed metabolites. Download Text S1, PDF file, 0.1 MB. Copyright © 2021 Mullins et al. 2021 Mullins et al. https://creativecommons.org/licenses/by/4.0/ This content is distributed under the terms of the Creative Commons Attribution 4.0 International license.

10.1128/mBio.00715-21.8 TABLE S3 PCR primers used for P. protegens clean deletion mutagenesis. Download Table S3, PDF file, 0.1 MB. Copyright © 2021 Mullins et al. 2021 Mullins et al. https://creativecommons.org/licenses/by/4.0/ This content is distributed under the terms of the Creative Commons Attribution 4.0 International license.

10.1128/mBio.00715-21.9 TABLE S4 PCR primers used for P. protegens and *T. caryophylli* gene replacement mutagenesis. Download Table S4, PDF file, 0.04 MB. Copyright © 2021 Mullins et al. 2021 Mullins et al. https://creativecommons.org/licenses/by/4.0/ This content is distributed under the terms of the Creative Commons Attribution 4.0 International license.
