# Supplementary figures and images for: Discovery of the Pseudomonas Polyyne Protegencin by a Phylogeny-Guided Study of Polyyne Biosynthetic Gene Cluster Diversity
Source: mBio. 2021 Aug 3;12(4):e00715-21. doi: 10.1128/mBio.00715-21 (PMC8406139; doi:10.1128/mBio.00715-21)

Fig. S1

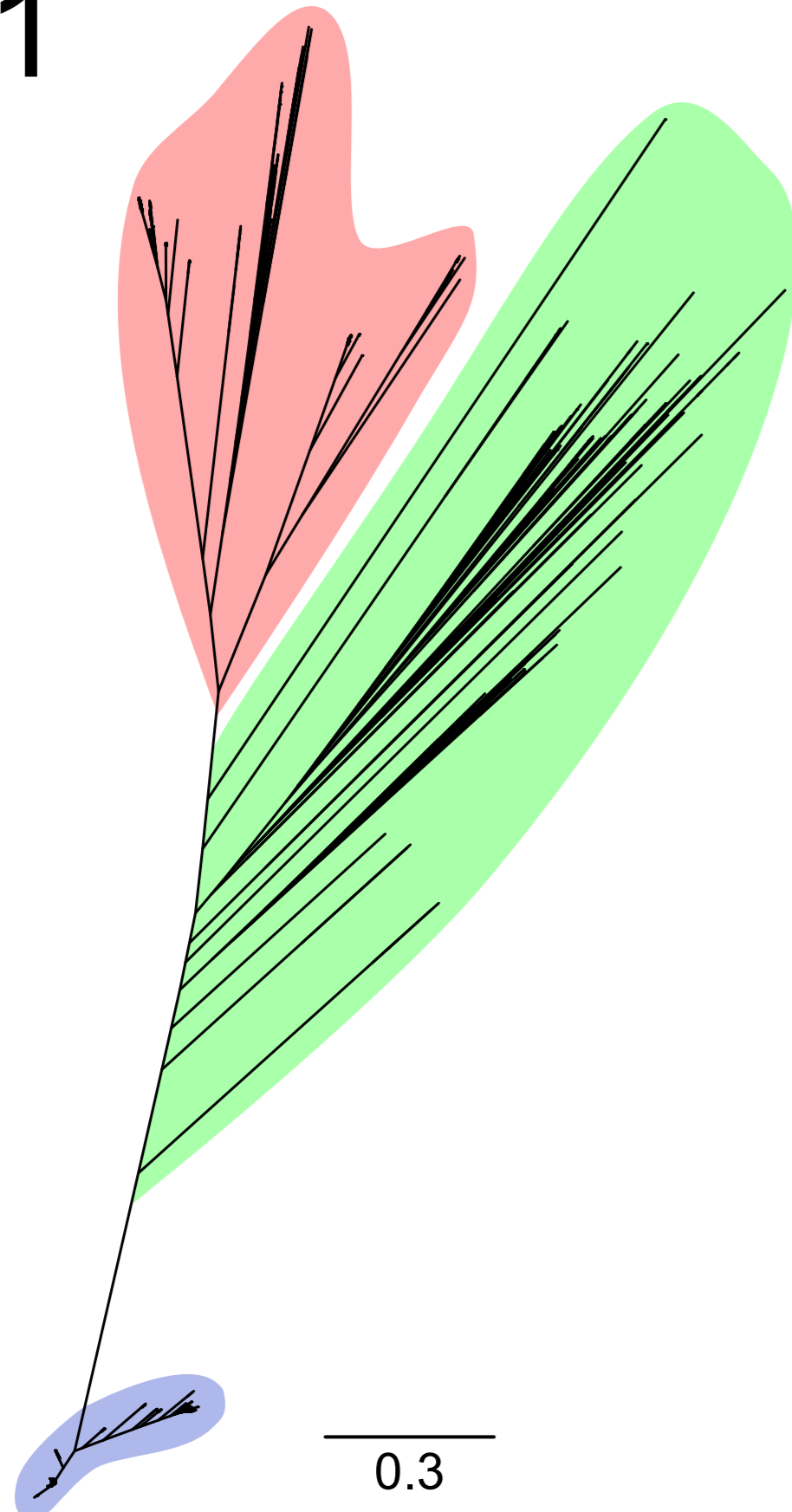

**a**

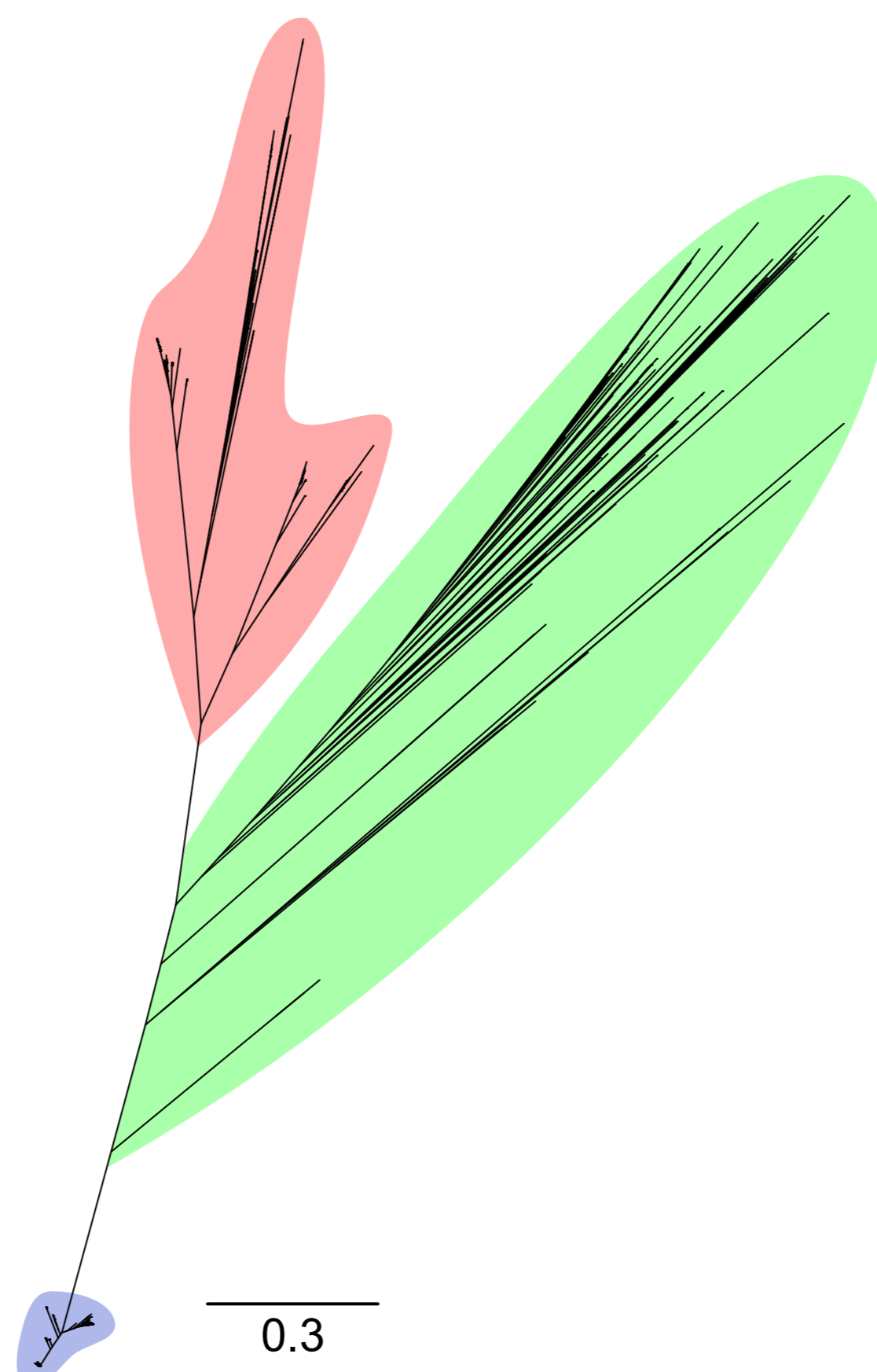

**b**

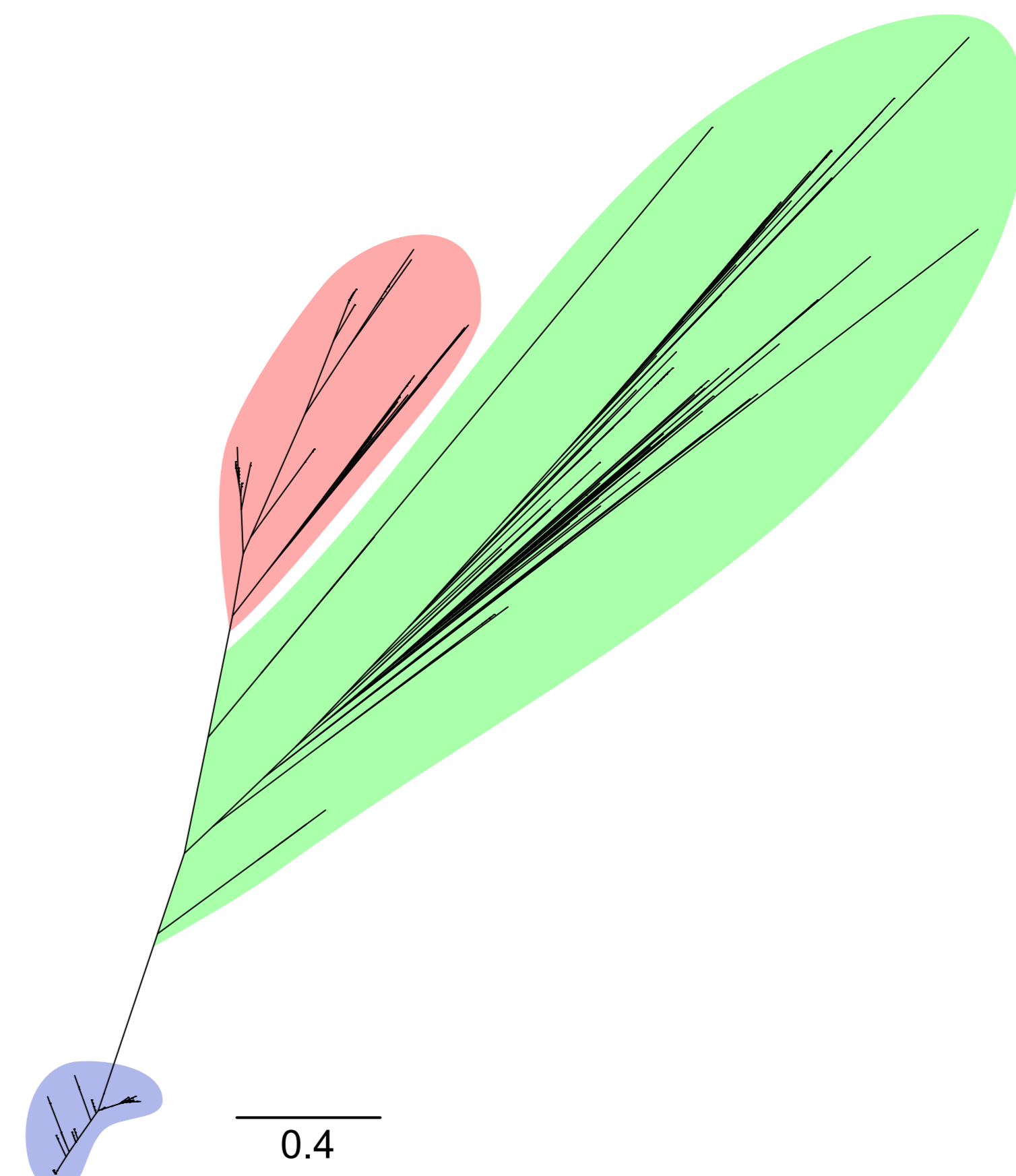

**c**

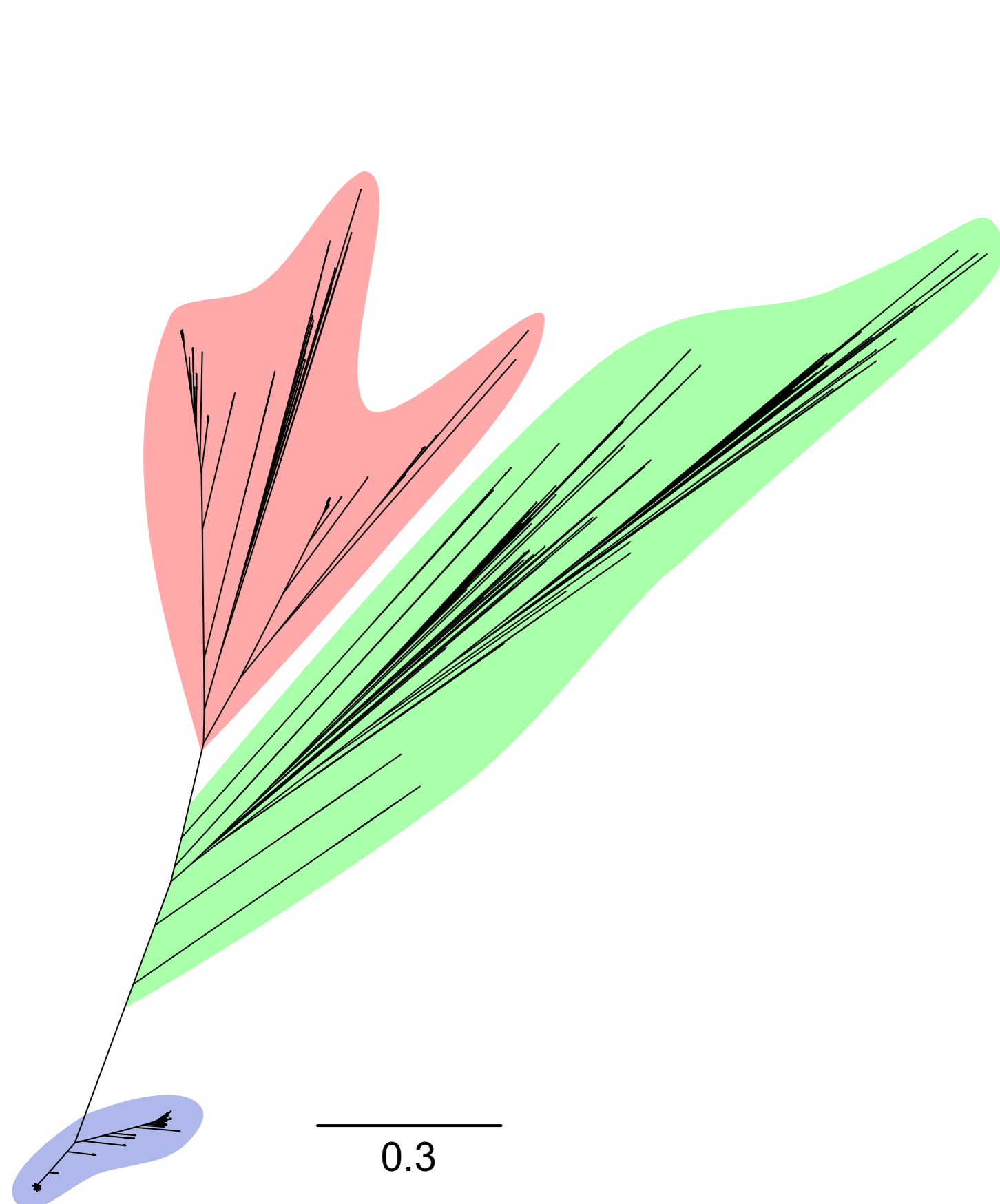

**d**

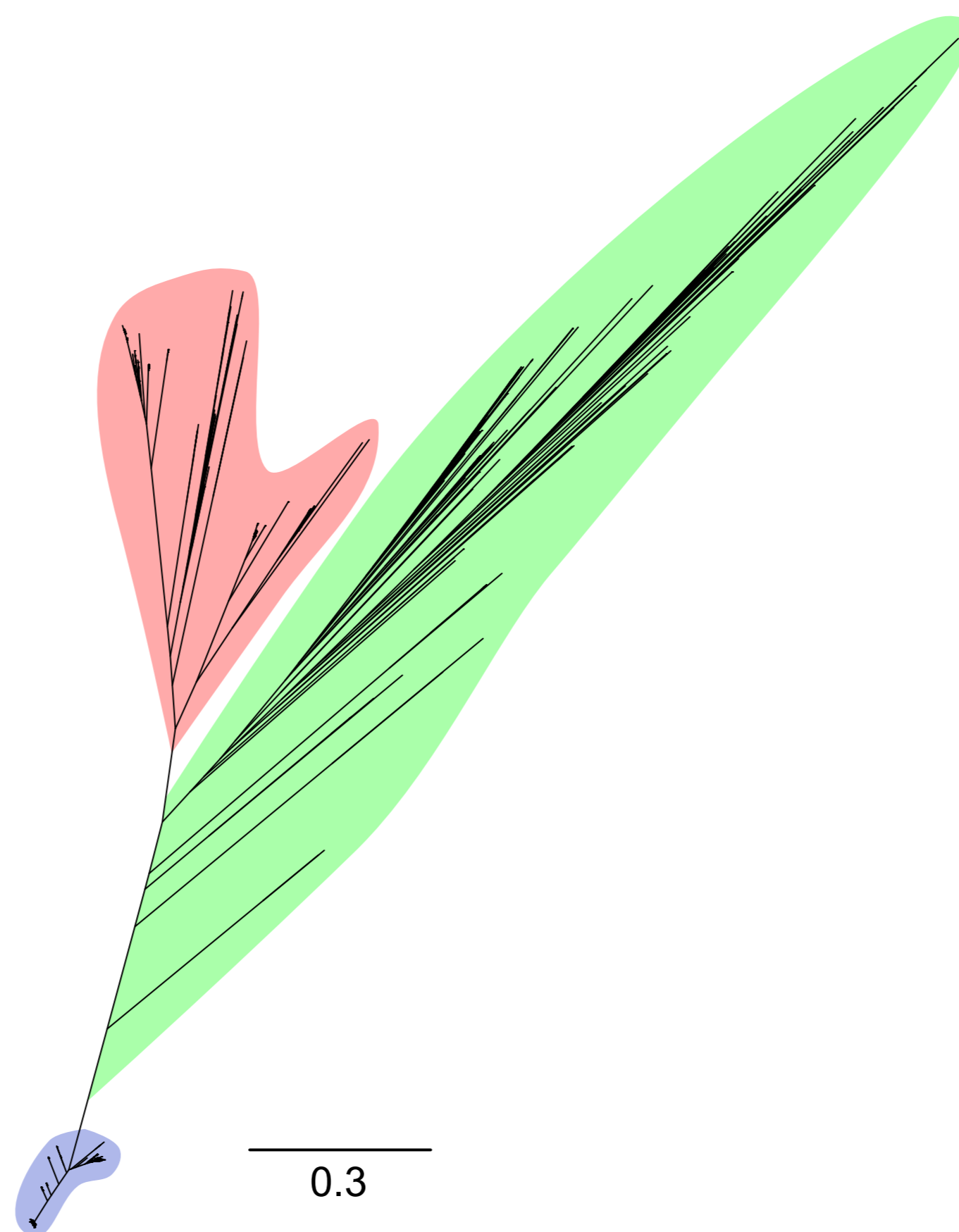

**e**

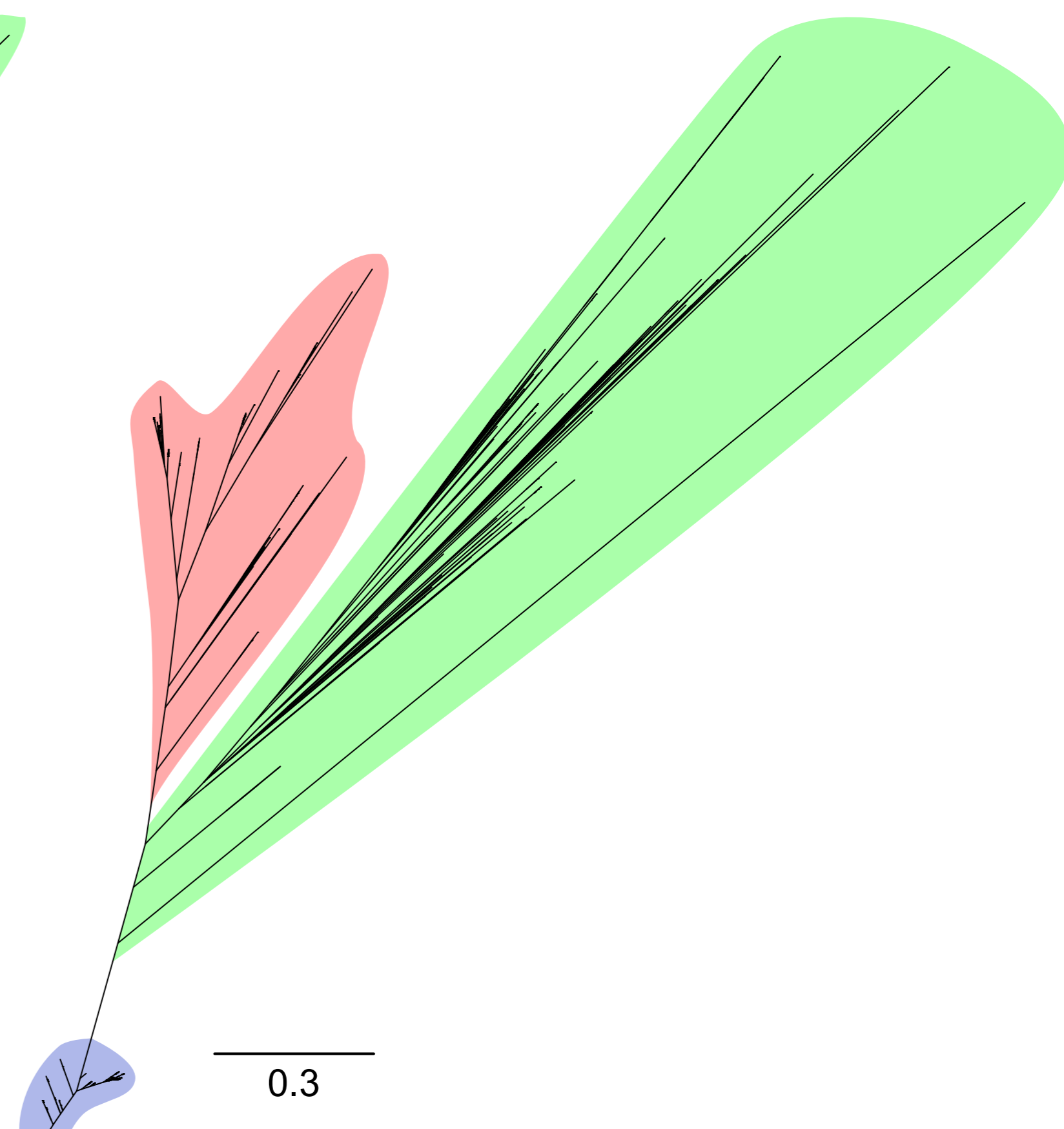

**f**

Supplement: FIG S1 [file mbio.00715-21-sf001.pdf]

Fig. S2

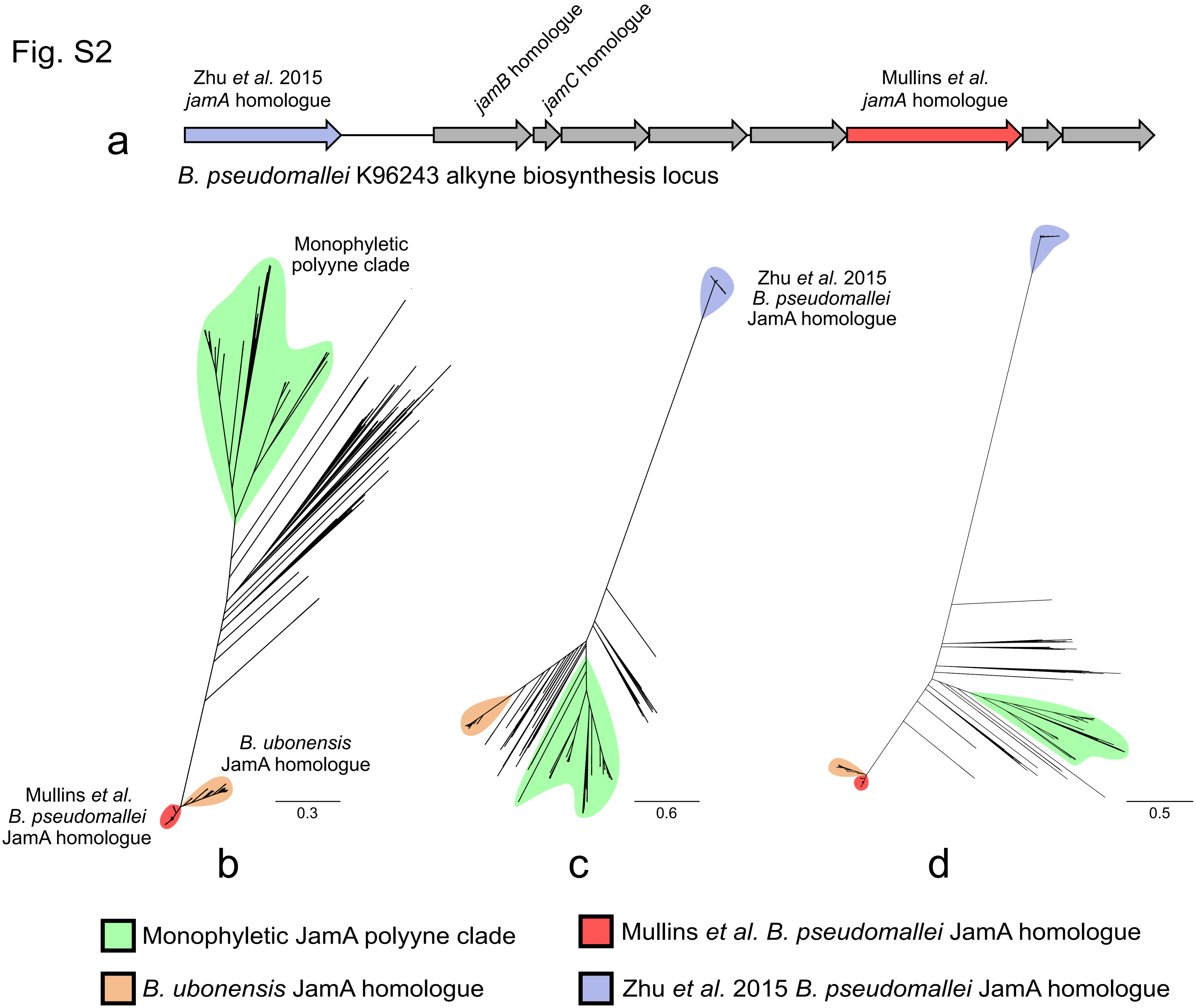

Supplement: FIG S2 [file mbio.00715-21-sf002.pdf]

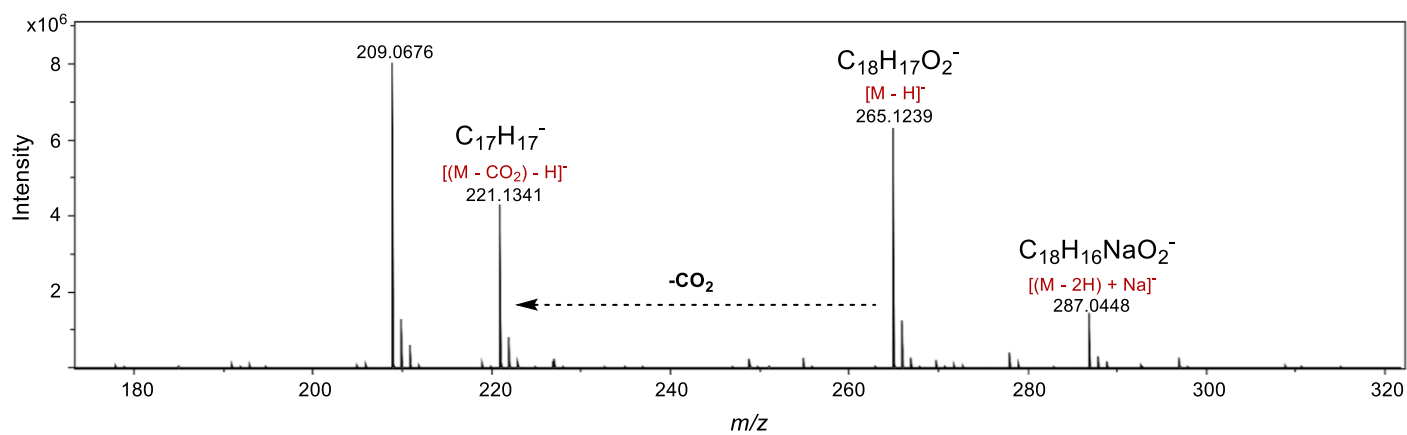

**Fig. S3a**

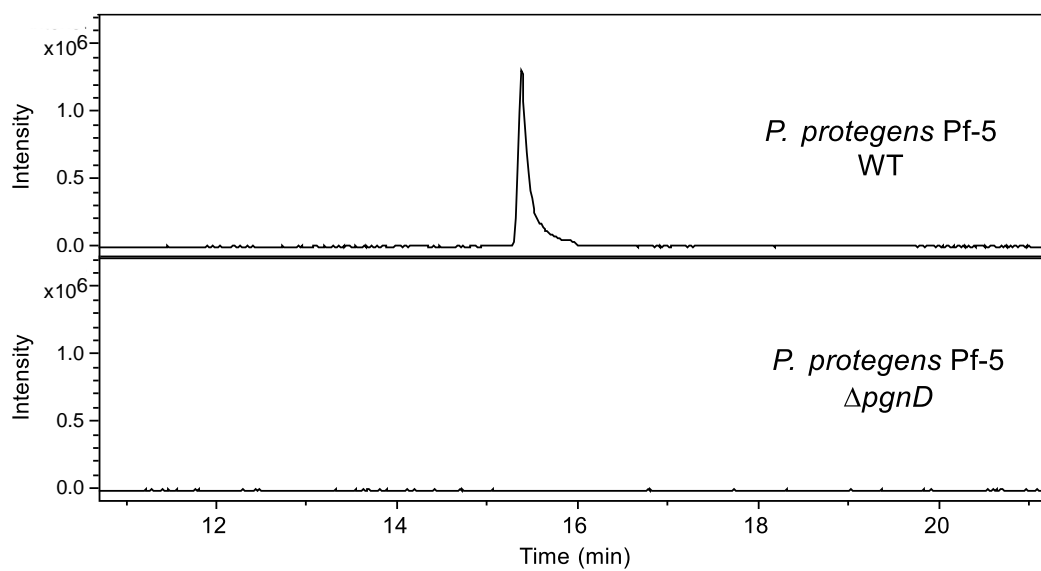

**Fig. S3b**

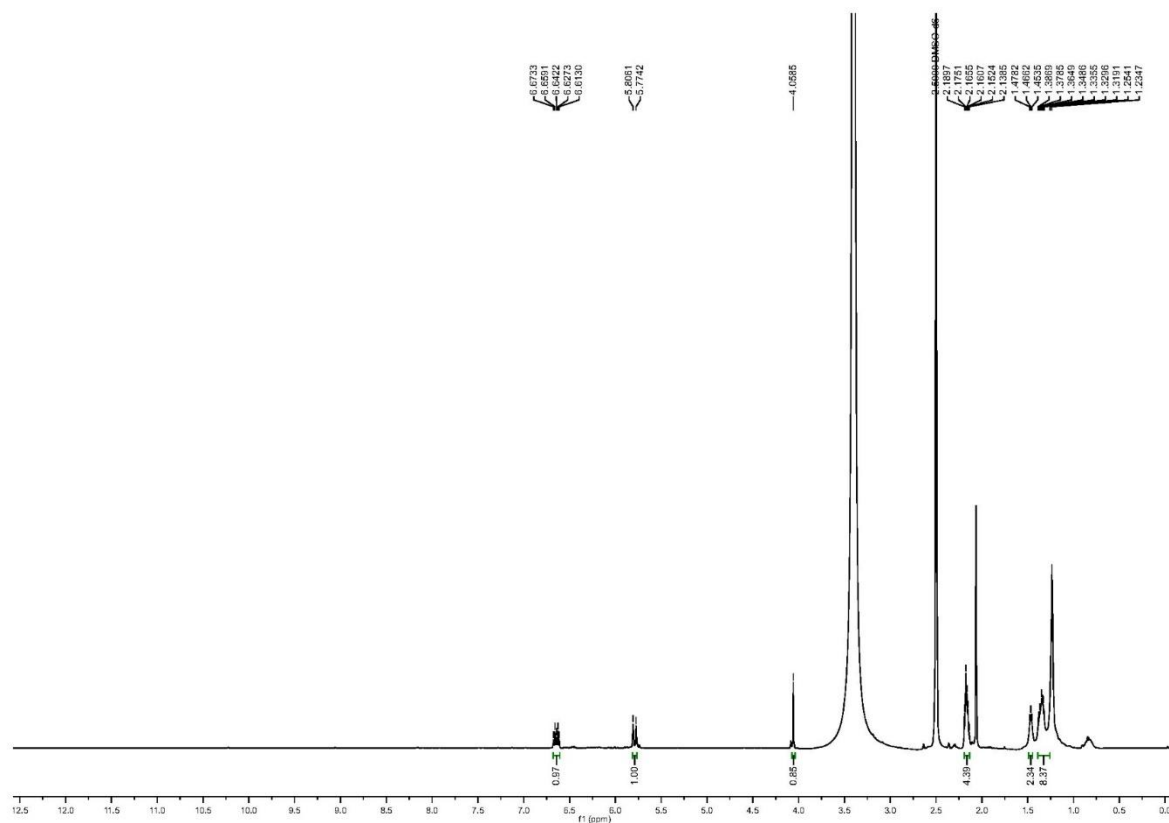

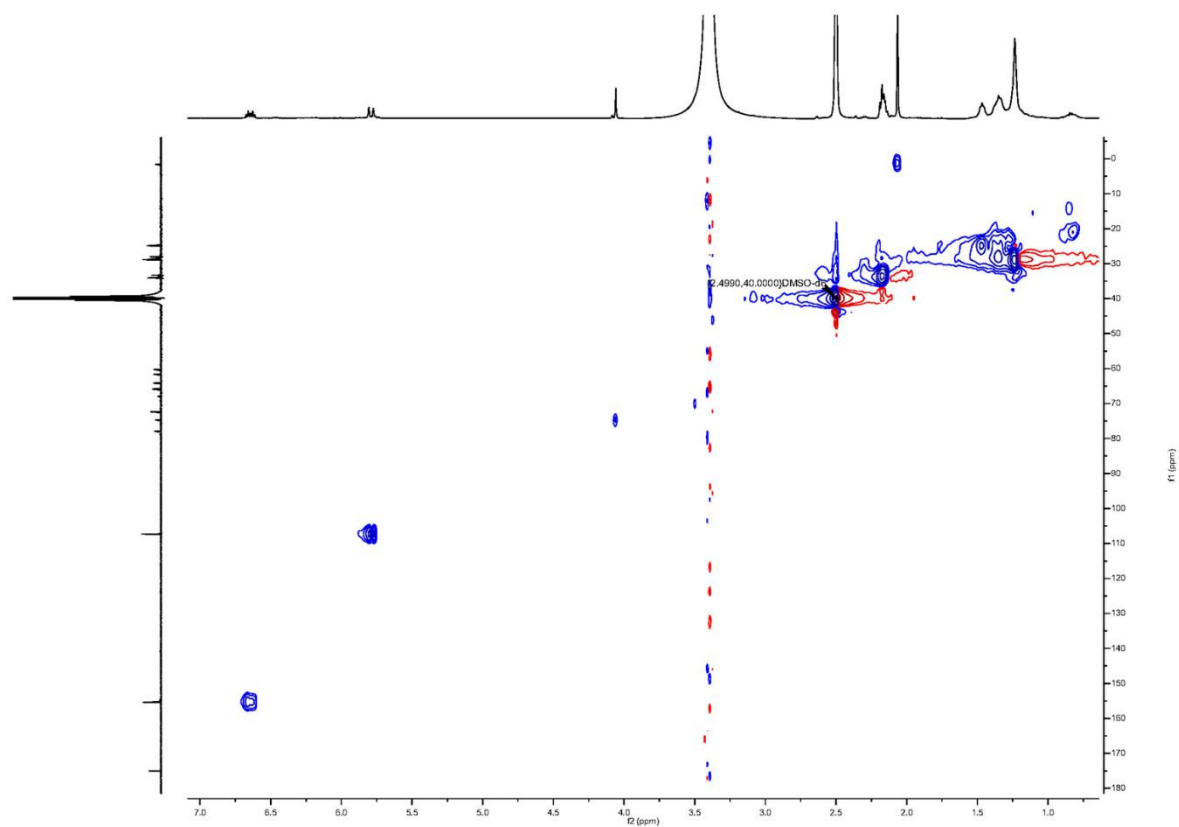

Fig. S3e

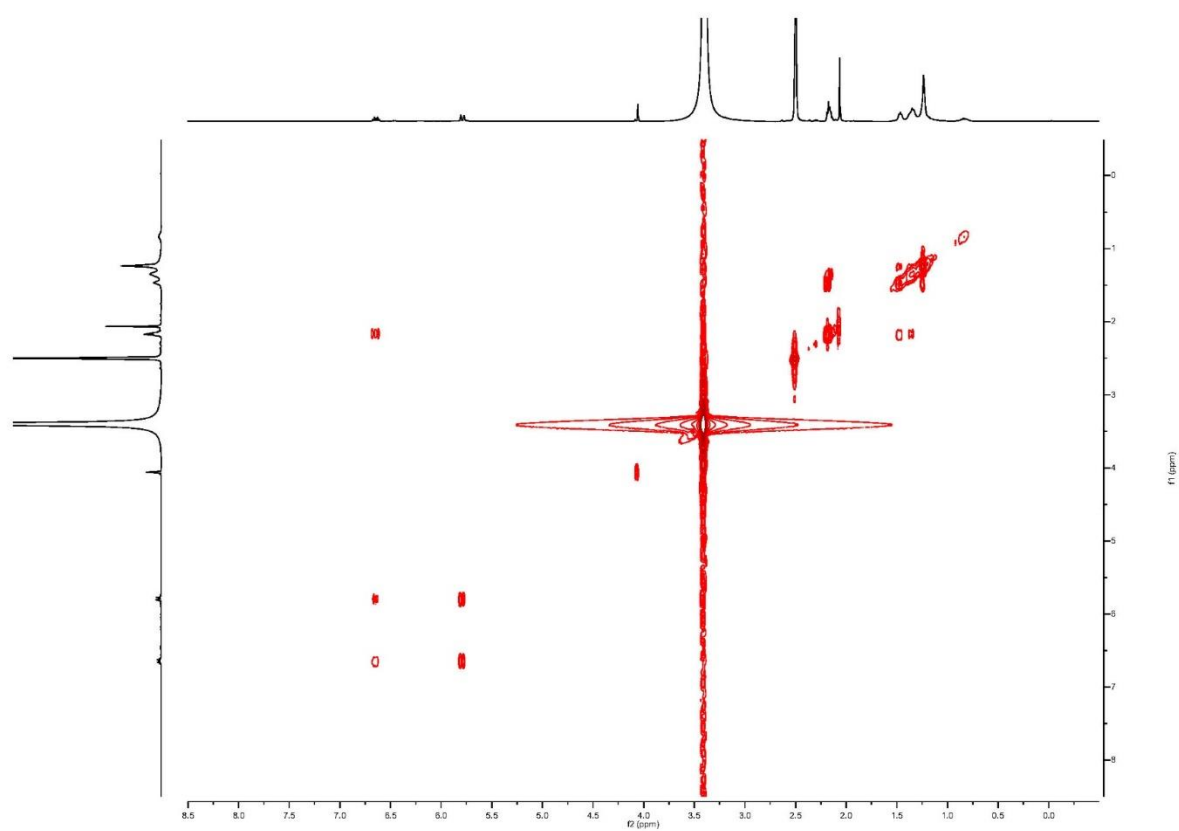

Fig. S3f

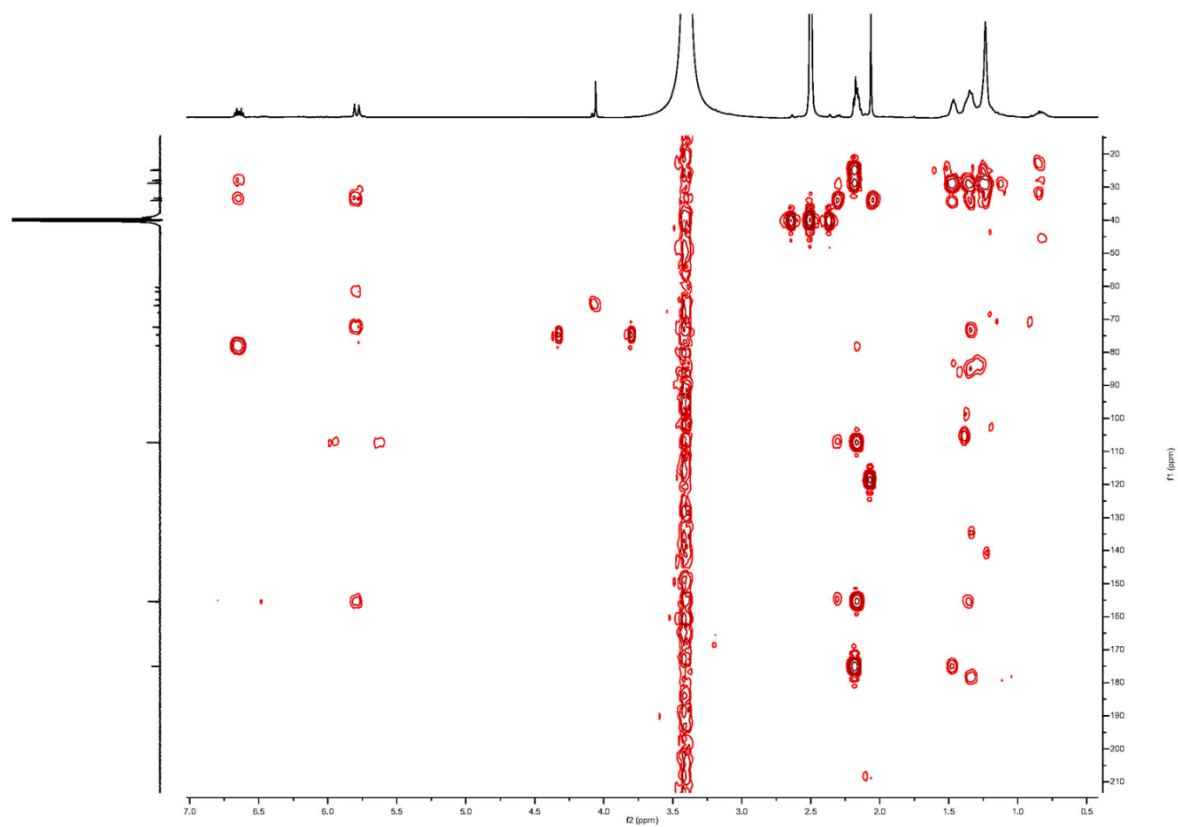

Fig. S3g

Supplement: FIG S3 [file mbio.00715-21-sf003.pdf]

Fig. S4

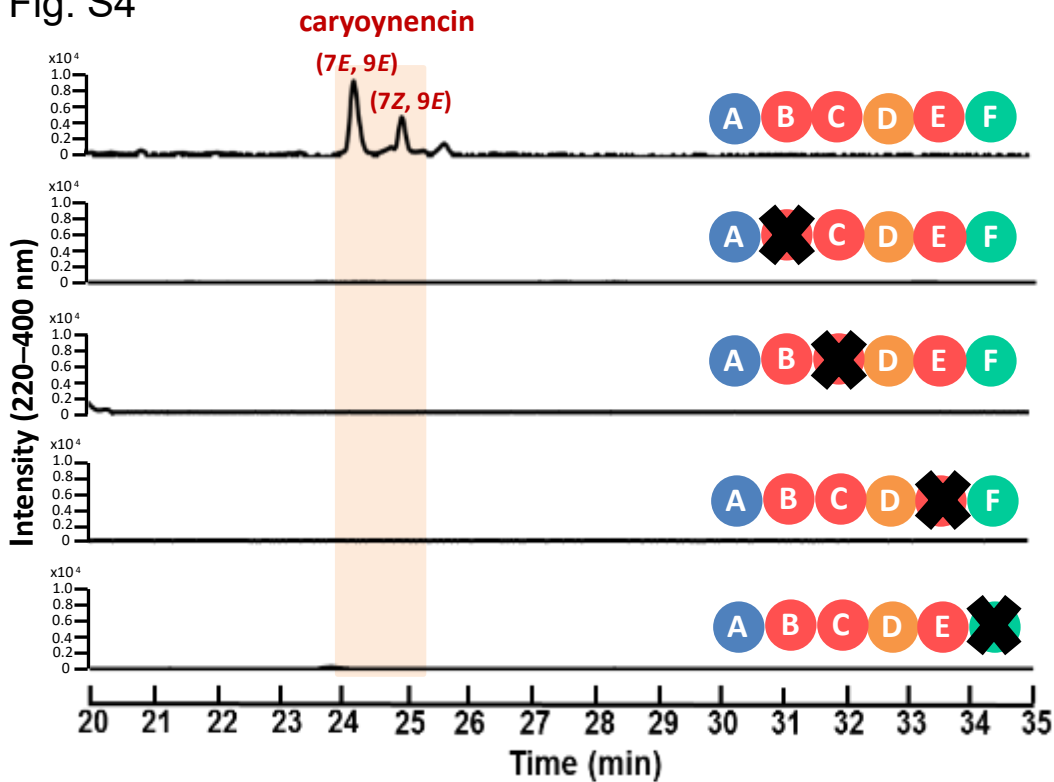

Supplement: FIG S4 [file mbio.00715-21-sf004.pdf]
