# Supplementary material for: Discovery of the Pseudomonas Polyyne Protegencin by a Phylogeny-Guided Study of Polyyne Biosynthetic Gene Cluster Diversity
Source: mBio. 2021 Aug 3;12(4):e00715-21. doi: 10.1128/mBio.00715-21 (PMC8406139; doi:10.1128/mBio.00715-21)
Supplement: TABLE S1 [file mbio.00715-21-st001.pdf]

Table S1. Strains and plasmids used in this study for mutagenesis.

Strains/Plasmids	Function	Source or Reference
**Strain**		
Escherichia coli		
DH5α	Tri-parental conjugation donor strain	Invitrogen
HB101 pRK2013	Tri-parental conjugation helper strain	Figurski and Helinski (1979) [14]
E. coli TOP10	Competent cells for bulk preparation of pJET1.2- Apr^R	Invitrogen
E. coli XL1-Blue	Competent cells for bulk preparation of pJET1.2- Apr^R	Invitrogen
Saccharomyces cerevisiae		
YPH500 (ATCC 76626)	Homologous recombination of allelic exchange vector	Pahirulzaman *et al.* (2012) [15]
Pseudomonas protegens		
CHA0 (DSM 19095^T)	Wild-type for mutagenesis	German Collection of Microorganisms and Cell Cultures (DSMZ)
Pf-5 (ATCC BAA-477)	Wild-type for mutagenesis	Howell and Stipanovic.(1979) [16]
CHA0Δ*pgnD*	Mutant with clean deletion of fatty acyl-AMP ligase, *pgnD*	This study
Pf-5Δ*pgnD*	Mutant with clean deletion of fatty acyl-AMP ligase, *pgnD*	This study
Pf-5Δ*pgnE*-Kan^R	*pgnE* gene replacement mutant with Kan^R cassette	This study
Pf-5Δ*pgnF*-Kan^R	*pgnF* gene replacement mutant with Kan^R cassette	This study
Pf-5Δ*pgnH*-Kan^R	*pgnH* gene replacement mutant with Kan^R cassette	This study
T. caryophylli		
Wild type (DSM50341)	Wild-type for mutagenesis	German Collection of Microorganisms and Cell Cultures (DSMZ)
Δ*cayB*-Apr^R	*cayB* gene replacement mutant with Apr^R cassette	This study
Δ*cayC*-Apr^R	*cayC* gene replacement mutant with Apr^R cassette	This study
Δ*cayE*-Apr^R	*cayE* gene replacement mutant with Apr^R cassette	This study
Δ*cayF*-Apr^R	*cayF* gene replacement mutant with Apr^R cassette	This study
**Plasmids**		
pMQ30	Allelic exchange vector	Shanks *et al.* (2006) [17]
pMQ30_F1_F2	Recombinant allelic exchange vector with *pgnD* flanking homology arms	This study
pGEM-Kan	Source of kanamycin resistance gene (Kan^R)	Ishida, Lincke and Hertweck (2012) [18]
PIJ773	Source of apramycin resistance gene (Apr^R)	Gust *et al*. (2003) [19]
pJET1.2	Introduce Apr^R to target organism	Thermo Fisher Scientific
pGL42a	Introduce Kan^R to target organism	Lackner, Moebius and Hertweck (2011) [20]
