# Supplementary material for: Discovery of the Pseudomonas Polyyne Protegencin by a Phylogeny-Guided Study of Polyyne Biosynthetic Gene Cluster Diversity
Source: mBio. 2021 Aug 3;12(4):e00715-21. doi: 10.1128/mBio.00715-21 (PMC8406139; doi:10.1128/mBio.00715-21)
Supplement: TABLE S2 [file mbio.00715-21-st002.pdf]

Table S2. ^{1}H **(500 MHz) and** ^{13}C **(125 MHz) NMR spectroscopic data of protegencin.**

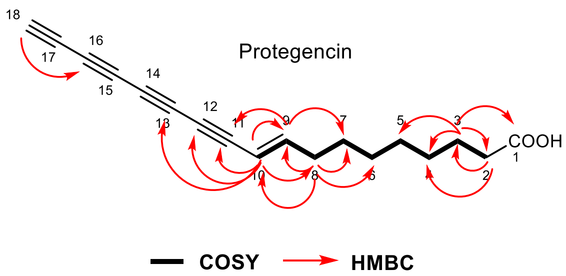

Position	δ_H (*J* in Hz)	δ_C, type
1		175.0, C
2	2.18,overlapped	34.1, CH_2
3	1.47, t-like (6.0, 6.5)	24.9, CH_2
4	1.25, overlapped	28.9, CH_2
5	1.35, overlapped	28.8, CH_2
6	1.34, overlapped	28.9, CH_2
7	1.35, overlapped	28.0, CH_2
8	2.15, overlapped	33.4, CH_2
9	6.65, dt (16.0, 6.5)	155.4, CH
10	5.79, d (16.0)	107.3, CH
11		78.0, C
12		72.3 C
13		61.6, C
14		65.8*, C
15		60.3*, C
16		64.2, C
17		72.3*, C
18	4.06, s	74.7, CH

*Might be interchanged.
