# Supplementary material for: Discovery of the Pseudomonas Polyyne Protegencin by a Phylogeny-Guided Study of Polyyne Biosynthetic Gene Cluster Diversity
Source: mBio. 2021 Aug 3;12(4):e00715-21. doi: 10.1128/mBio.00715-21 (PMC8406139; doi:10.1128/mBio.00715-21)
Supplement: TEXT S1 [file mbio.00715-21-t0001.pdf]

Supplementary Information

Discovery of the *Pseudomonas* polyyne protegencin by phylogeny-guided study of polyyne biosynthetic gene cluster diversity

Alex J. Mullins[a†*], Gordon Webster[a†], Hak Joong Kim[b†], Jinlian Zhao[c], Yoana D. Petrova[a], Christina E. Ramming[c], Matthew Jenner[c,d], James A. H. Murray[e], Thomas R. Connor[a], Christian Hertweck[b,f], Gregory L. Challis[c,d,g,h] and Eshwar Mahenthiralingam[a*]

[†]Equal contribution

[a]Microbiomes, Microbes and Informatics Group, Organisms and Environment Division, School of Biosciences, Cardiff University, Cardiff, CF10 3AX, UK.

[b]Department of Biomolecular Chemistry, Leibniz Institute for Natural Product Research and Infection Biology, Hans Knöll Institute, Beutenbergstrasse 11a, 07745, Jena, Germany.

[c]Department of Chemistry, University of Warwick, Coventry, CV4 7AL, UK.

[d]Warwick Integrative Synthetic Biology Centre, University of Warwick, Coventry CV4 7AL, UK.

[e]Molecular Biosciences Division, School of Biosciences, Cardiff University, Cardiff, CF10 3AX, UK.

[f]Faculty of Biological Sciences, Friedrich Schiller University Jena, 07743 Jena, Germany.

[g]Department of Biochemistry and Molecular Biology, Biomedicine Discovery Institute, Monash University, Clayton, VIC 3800, Australia.

[h]ARC Centre of Excellence for Innovations in Peptide and Protein Science, Monash University, Clayton VIC 3800, Australia.

Supplementary Methods

In-frame deletion of fatty acyl-AMP ligase encoding-gene, *pgnD*, in *Pseudomonas protegens*

In brief, two fragments of approximately 500 bp flanking the fatty acyl-AMP ligase encoding-gene, *pgnD* (**Fig. 5a**), were PCR-amplified (**Table S3**) using Q5 DNA polymerase (NEB). The suicide vector pMQ30 was linearised by *EcoRI*/*HindIII* restriction digest (ThermoFisher) and co-transformed with the two PCR fragments into *Saccharomyces cerevisiae* YPH500 using the lithium acetate transformation method (1). Overlapping homologous 30 bp regions between the PCR fragments and linearised pMQ30 vector enabled yeast-mediated homologous recombination. The yeast transformation mixture was then grown on synthetic defined agar with 2% w/v D-glucose and 7.7 g/L complete supplement uracil dropout mixture (Formedium) for 3 – 4 d at 30 °C. Plasmid extraction was performed with Zymoprep Yeast Plasmid Miniprep I (Zymo Research), and transformed into *Escherichia coli* DH5α with selection on LB agar supplemented with 10 µg/ml gentamicin. Colonies were screened by PCR (**Table S3**) for the correct allelic exchange vector and confirmed by Sanger sequencing (Eurofins Genomics). The recombinant allelic exchange vector was introduced to *P. protegens* via tri-parental mating using the *E. coli* helper strain HB101 carrying pRK2013. Transconjugants were selected by growth on M9 medium supplemented with 2% (w/v) sodium citrate and 25 µg/ml gentamicin, and incubation for 36 – 48 h at 37 °C. Transconjugants were confirmed by PCR (**Table S3**) to detect recombination in either the upstream or downstream region. A second recombination event was triggered by sucrose counter-selection by growth on non-salt lysogeny broth with 15% (w/v) sucrose at 30 °C. Loss of the integrated allelic exchange vector was confirmed by loss of gentamicin resistance. PCR targeting the fatty acyl-AMP ligase encoding-gene, *pgnD* was used to distinguish between *P. protegens* wild-type and mutants, and later confirmed by Sanger sequencing (Eurofins Genomics).

Q5-based PCR (NEB) thermal cycler conditions were as follows: Initial denaturation at 98 °C for 30 s; 40x cycles of denaturation at 98 °C for 10 s, annealing at variable temperature (**Table S3**) for 30 s, and extension at 72 °C for 60 s; then a final extension at 72 °C for 2 min. *Pseudomonas* genomic DNA was extracted using the Maxwell 16® instrument and tissue DNA extraction kit (Promega, UK). *Taq*-based PCR (ThermoFisher) thermal cycler conditions were as follows: Initial denaturation at 95 °C for 5 min; 35x cycles of denaturation at 95 °C for 30 s, annealing at variable temperature (**Table S3**) for 30 s, and extension at 72 °C for 2 min; then a final extension at 72 °C for 10 min. Genomic DNA was extracted using 5% (w/v) Chelex 100 resin (2).

Construction and analysis of *P. protegens* Pf-5 gene replacement mutants

P. protegens Pf-5 was cultivated on Luria-Bertani (LB) agar at 30 °C for 2 d and on LB broth at 37 °C with orbital shaking (150 rpm) for 1 d. Genomic DNA from pure culture of *P. protegens* Pf-5 was acquired using MasterPure™ DNA purification kit. A targeted double-crossover strategy was chosen to insert the kanamycin resistance gene amplified from pGEM-Kan between the flank regions of each gene to inactivate *pgnE*, *pgnF*, and *pgnH*. The corresponding genes in the putative protegencin BGC were PCR-amplified from the genomic DNA of *P. protegens* Pf-5 and ligated with the kanamycin resistance gene. The combined gene fragment was inserted into the pGL42a vector by T4 DNA ligase. *E. coli* TOP 10 cells were transformed with the above vector through electrophoresis at 2,250V, and selected through LB plates supplemented with kanamycin (50 µg/ml). After inoculation of selected *E. coli* cells in LB media with kanamycin, the vector containing the combined gene fragment was purified by using Monarch® Plasmid Miniprep Kit from NEB. The vector was introduced into *P. protegens* Pf-5 by electroporation at 2,500 V, and the transformant was incubated on LB agar containing kanamycin at 30 °C. The successful integration of the kanamycin resistance cassette was verified with specific primers (**Table S4**). Verified colonies were inoculated into LB medium with kanamycin at 30 °C until the $OD_{600\ nm}$ value reached 4–5. Then, the bacterial cells were collected by centrifugation (5,000 rpm, 5 min) and washed with Tris-Acetate-

Phosphate (TAP) medium twice. *P. protegens* Pf-5 cells were incubated in TAP medium with kanamycin at 30 °C with orbital shaking (120 rpm) for 1 d.

Analytical HPLC was performed on a Shimadzu Prominence HPLC system consisting of an autosampler, high-pressure pumps, column oven and PDA using a Macherey-Nagel C18 reverse phase column (Nucleosil 100, 5 µm, 125 × 4.6 mm, flow rate 1 ml/min). HPLC-grade CH_3CN and deionized water with 0.1% trifluoroacetic acid were used as mobile phase for HPLC. The gradient elution was CH_3CH/H_2O with 0.1% (v/v) TFA 0.5/99.5 to 100/0 for 30 min, CH_3CN 100% for 10 min. Preparative HPLC was performed on a Gilson Abimed equipped with Binary Pump 321 and 156 UV/Vis detector (eluent: water with 0.1% (v/v) TFA, acetonitrile) using a Macherey-Nagel C18 reverse phase column (Nucleosil 100, 5 µm, 250 × 10 mm, flow rate 5 ml/min). LC-MS measurements were performed using a QExactive Orbitrap High Performance Benchtop LC-MS with an electrospray ion source and an Accela HPLC system (Thermo Fisher Scientific, Bremen). For MS/MS measurements an Exactive Orbitrap mass spectrometer with an electrospray ion source (Thermo Fisher Scientific, Bremen).

Construction and analysis of *T. caryophylli* gene replacement mutants

A targeted double crossover strategy was chosen to insert the apramycin resistant gene cassette from pIJ773 between the flank regions of each gene to inactivate the *cayB*, *cayC*, *cayE* and *cayF*. Each gene in the *cay* gene cluster of was PCR-amplified from the genomic DNA of *T. caryophylli* and ligated with the apramycin resistance gene. The combined gene fragment was inserted into the pJET1.2/blunt vector and introduced into XL1-Blue *E. coli* competent cells. After inoculating of *E. coli* cells in LB media with apramycin and ampicillin selection markers, the vector containing the combined gene fragment was purified using the Monarch® Plasmid Miniprep Kit from NEB. The vector was introduced into *T. caryophylli* by electroporation at 2,500 V, and the transformant was incubated on potato dextrose agar (PDA) plates containing apramycin and ampicillin at 30 °C. Successful integration of the apramycin resistance cassette was verified with specific primers (**Table S4**). The verified colonies were

inoculated into potato dextrose broth with apramycin and ampicillin at 30 °C for 1 d. The culture was incubated on potato dextrose agar with apramycin and ampicillin selection for additional 4 d at 30 °C. Ethyl acetate was added into the small pieces cut from the agar plate and kept for 2 h. The ethyl acetate extract was filtered and concentrated under reduced pressure. The analytic methods were same as *P. protegens* Pf-5 mutation studies.

Pea exudate medium (PEM) preparation

An agar composed of pea (*Pisum sativum*) seed exudate was prepared as a growth condition representative of the nutrient availability surrounding a germinating seed. Approximately 100 g of Early Onward variety *P. sativum* seeds were rinsed three times with de-ionised water, and finally suspended in distilled water made up to 500 ml. The seeds were incubated in the dark with agitation (40 rpm on a rocking platform) for 2 d at 22 °C. The seed exudate was removed and filtered twice, initially with a grade GF/D glass microfibre filter, and finally a grade GF/A glass microfibre filter. The filtered seed exudate was combined with distilled water at a 1:1 ratio, and purified agar (Oxoid) added to make a 1.5% agar when autoclaved.

References

1. Pahirulzaman KAK, Williams K, Lazarus CM. 2012. A toolkit for heterologous expression of metabolic pathways in *Aspergillus oryzae*, p. 241–260. *In* Methods in Enzymology. Academic Press Inc.

2. Walsh PS, Metzger DA, Higuchi R. 2013. Chelex 100 as a Medium for Simple Extraction of DNA for PCR-Based Typing from Forensic Material. Biotechniques 54:134–139.
