# Supplementary material for: Discovery of the Pseudomonas Polyyne Protegencin by a Phylogeny-Guided Study of Polyyne Biosynthetic Gene Cluster Diversity
Source: mBio. 2021 Aug 3;12(4):e00715-21. doi: 10.1128/mBio.00715-21 (PMC8406139; doi:10.1128/mBio.00715-21)
Supplement: TABLE S3 [file mbio.00715-21-st003.pdf]

Table S3. PCR primers used for *P. protegens* clean deletion mutagenesis.

Primers	Final product size (bp)	Primer use	Ta (°C)[a]/DNA polymerase
**F1_fwd:** 5'-CACGACGTTGTAAAACGACGGCCAGTGCCAGGCCTGGACCTTGTCCAGTG-3'<br>**F1_rev:** 5'-TGGCCGTCATGACAGGTTCATTCAAAATCCTTTTTAAATGGAGAGCC-3'	545	Amplify upstream fragment (F1)	62.5 / Q5
**F2_fwd:** 5'-GGATTTTGAATGAACCTGTCATGACGGCCATGAATC-3'<br>**F2_rev:** 5'-ACACAGGAAACAGCTATGACCATGATTACGATCTGCATGATCAGTCGATC-3'	550	Amplify downstream fragment (F2)	65 / Q5
**M13_fwd:** 5'-AGGGTTTTCCCAGTCACGACGTT-3'<br>**M13_rev:** 5'-GAGCGGATAACAATTTCACACAGG-3'	1,090 (pMQ30_F1_F2)<br>146 in (pMQ30)	Confirm recombinant pMQ30_F1_F2 construct	58 / *Taq*
**M13_fwd:** 5'-AGGGTTTTCCCAGTCACGACGTT-3'<br>**Seq_chr_rev:** 5'-TACCGTCGGAATCGCCAGCC-3'	3,132<br>(recombination at F1)<br>1,284<br>(recombination at F2)	Confirm transconjugant *P. protegens*	60 / *Taq*
**Seq_chr_fwd:** 5'-CAGGACGAAAACTGCTGAACAGGA-3'<br>**M13_rev:** 5'-GAGCGGATAACAATTTCACACAGG-3'	1,271<br>(recombination at F1)<br>3,119<br>(recombination at F2)	Confirm transconjugant *P. protegens*	60 / *Taq*
**Seq_chr_fwd:** 5'-CAGGACGAAAACTGCTGAACAGGA-3'<br>**Seq_chr_rev:** 5'- TACCGTCGGAATCGCCAGC C-3'	3,313 (wild-type)<br>1,465 (clean deletion)	Confirm *pgnD* clean deletion	62 / *Taq*

[a]Ta = Annealing temperature
