# Supplementary material for: Discovery of the Pseudomonas Polyyne Protegencin by a Phylogeny-Guided Study of Polyyne Biosynthetic Gene Cluster Diversity
Source: mBio. 2021 Aug 3;12(4):e00715-21. doi: 10.1128/mBio.00715-21 (PMC8406139; doi:10.1128/mBio.00715-21)
Supplement: TABLE S4 [file mbio.00715-21-st004.pdf]

Table S4. PCR primers used for *P. protegens* and *T. caryophylli* gene replacement mutagenesis[a].

P. protegens		
**Gene**	**Forward primer**	**Reverse primer**
pgnE Fl1	5'- AAA AAA CTA GTG GCT GAC GGT CGC AAC TCC-3'	5'- GCT ACT TAA TTA AGC TAG CGT AGC CCC TGG ATA TTG CCG ATA AA-3'
pgnE Fl2	5'-GCT ACG CTA GCT TAA TTA AGT AGC ATA ACC CGC AGC TGG GAG-3'	5'- AAA AAG AGC TCC AGA AGA ACT CGT CAA GAA GGC G-3'
pgnF Fl1	5'- AAA AAA CTA GTA CAC AGC GAC ACC AGA GGT C-3'	5'-GCT ACT TAA TTA AGC TAG CGT AGC ATC AGC ATC AGG CTG CTC ATC-3'
pgnF Fl2	5'-GCT ACG CTA GCT TAA TTA AGT AGC ATC TTC GTC GCC AAC CAG GCG-3'	5'- AAA AAG AGC TCT GCG GAC TTT GTC GAT CAT TG-3'
pgnH Fl1	5'- AAA AAA CTA GTA CGC TCT CAG GAG ATC GAG GG-3'	5'-GCT ACT TAA TTA AGC TAG CGT AGC TGC ATG TTG TGC ATG TGG C-3'
pgnH Fl2	5'-GCT ACG CTA GCT TAA TTA AGT AGC AAC GGC TAC CTG AAG CTC-3'	5'- AAA AAG AGC TCT TCG GCC ACC TTG ACG TTC TC -3'
Kan^R	5'-GCT ACG CTA GCG TAA GCT TAG GCT GCT GCC-3'	5'-GCT ACT TAA TTA ATC AGA AA ACT CGT CAA GAA GGC G -3'
T. caryophylli		
**Gene**	**Forward primer**	**Reverse primer**
cayB Fl1	5'-ATC AGC ATC CGC ATG CGT C-3'	5'-GCT ACT TAA TTA AGC TAG CGT AGC GTC GCC TTC CTG ATC GGT-3'
cayB Fl2	5'-GCT ACG CTA GCT TAA TTA AGT AGC ATC GGC GGA ACT GCG TAC-3'	5'-GCT CGA TGA ACG AGC CGG A-3'
cayC Fl1	5'-ACG TAT CAA GGC GTT GAC CGC-3'	5'-GCT ACT TAA TTA AGC TAG CGT AGC ACC CAG AAT AGA ATC GGA C-3'
cayC Fl2	5'-GCT ACG CTA GCT TAA TTA AGT AGC ATT GCC AAT CAG TTC GCG-3'	5'-GAC GCG GTC GAT CAT GT-3'
cayE Fl1	5'-ATT CAG CTA CCC CGA CAG C-3'	5'-GCT ACT TAA TTA AGC TAG CGT AGC ATG CAG GTT ATG CAT GTG-3'
cayE Fl2	5'-GCT ACG CTA GCT TAA TTA AGT AGC ATC ACA TCT CGG ATG ATC-3'	5'-CGA CTG GTA ATA GCC GTG CA-3'
cayF Fl1	5'-ACA GAG CAG TCC ATT CTT CG-3'	5'-GCT ACT TAA TTA AGC TAG CGT AGC AAC ATC GTG CTG CTG TCG-3'
Apr^R	5'-GCT ACG CTA GCA TTC CGG GGA TCC GTC GAC C-3'	5'-GCT ACT TAA TTA ATG TAG GCT GGA GCT GCT TC-3'

[a]Restriction sites are underlined.
